# Predictive factors for the diagnosis of coeliac disease in children and young people in primary care: A systematic review and meta-analysis

**DOI:** 10.1371/journal.pone.0306844

**Published:** 2024-12-20

**Authors:** Christian E. Farrier, Marta Wanat, Anthony Harnden, Amy Paterson, Nia Roberts, Defne Saatci, Jennifer Hirst

**Affiliations:** 1 Nuffield Department of Primary Care Health Sciences, University of Oxford, Oxford, United Kingdom; 2 Department of Paediatrics, Cumming School of Medicine, University of Calgary, Calgary, AB, Canada; 3 Nuffield Department of Medicine, University of Oxford, Oxford, United Kingdom; 4 Bodleian Healthcare Libraries, Knowledge Centre, University of Oxford, Oxford, United Kingdom; Gaziantep Islam Science and Technology University, Medical Faculty, Division of Pediatric Gastroenterology, TÜRKIYE

## Abstract

**Background:**

Coeliac Disease (CD) often has its onset in childhood and affects 1% of the population. This review aimed to identify important predictive factors for coeliac disease in children and young people which could help GPs decide when to offer testing.

**Methods:**

We searched MEDLINE, Embase and Cochrane Library to April 2024. Included studies were observational or randomized trials reporting the risk of CD when exposed to predictive factor(s) in people ≤25 years of age. Genetic factors were excluded. Risk of Bias was assessed using the Newcastle-Ottawa Scale. Random effects meta-analysis was performed for factors reported in ≥5 studies to calculate pooled odds ratios (OR) or standardized mean differences (SMD).

**Results:**

Of 11,623 unique abstracts, 183 were included reporting on 140+ potentially associated factors. Meta-analyses of 28 factors found 14 significant associations with CD diagnosis: having type 1 diabetes (OR 8.70), having a first degree relative with coeliac disease (OR 5.19), being of white ethnicity (OR 2.56), having thyroid disease (OR 2.16), being female (OR 1.53), more frequent gastroenteritis in early childhood (OR 1.48), having frequent respiratory infections in early childhood (OR 1.47), more gluten ingestion in early life (OR 1.25), having more infections in early life (OR 1.22), antibiotic use in early childhood (OR 1.21), being born in the summer (OR 1.09), breastfeeding (OR 0.79) older age at diagnosis of type 1 diabetes (OR 0.64), and heavier weight (SMD -0.21). The final three were associated with lower risk of CD diagnosis.

**Discussion:**

This is the first systematic review and meta-analysis of predictive factors for CD in children. Amongst the 14 factors we identified that were significant, three were potentially modifiable: breast feeding, antibiotic use and amount of gluten ingestion in early childhood. This work could inform the development of clinical support tools to facilitate the early diagnosis of CD.

## Introduction

Coeliac Disease (CD) is a chronic immune-mediated enteropathy in response to dietary gluten. CD affects 1.4% of the population globally and similar disease burden has been reported in the UK [1–4]. Despite having a peak of onset in early childhood, CD is frequently underdiagnosed or missed in children because of its variable and nonspecific presentation [5, 6]. Delayed or missed diagnosis can lead to complications, including malnutrition, poor growth, osteoporosis, and intestinal damage [7]. CD is treated with a lifelong gluten free diet which can prevent long-term complications and allow healing of the intestinal mucosa [6].

The process of diagnosing CD begins with bloodwork, typically testing for anti-tissue transglutaminase (tTG) and/or anti-endomysial (EMA) immunoglobulin A (IgA) [8–10]. A serum IgA level is also assessed, because in IgA-deficient patients, tTG-IgA and EMA-IgA are not reliable, and the corresponding immunoglobulin G (IgG) tests are indicated [11]. Genetic testing and HLA-typing may be used to rule out CD in individuals without genetic susceptibility but are not sufficient to confirm a diagnosis of CD [10]. Historically, all patients required a confirmatory duodenal biopsy to establish the diagnosis of CD [8]. Recent European guidelines have allowed for the diagnosis of CD in children without a confirmatory biopsy when the anti-tTG is greater than ten times the upper limit of normal and there is a confirmatory anti-EMA on a subsequent blood sample [9, 10]. Patients not meeting these criteria but still with elevated anti-tTG levels would need to have a duodenal biopsy to confirm the diagnosis [9, 10].

Although there has been discussion about the potential role of a population screening program for CD, currently an active-case finding strategy is recommended with screening at-risk groups and using symptom-based testing [8–11]. For example, the UK NICE guidelines advise that serological testing should be offered to people with persistent or unexplained symptoms potentially consistent with CD, a family history of CD in first-degree, or high risk conditions such as Type 1 Diabetes Mellitus (T1DM) or autoimmune thyroid disease [11]. This is reaffirmed in recent published literature demonstrating the higher prevalence of CD amongst children with T1DM [12], autoimmune thyroid antibodies in children with CD [13], and the higher rates of CD amongst children with a sibling diagnosed with CD [14]. Appropriate screening and testing facilitate timely diagnosis and initiation of a gluten-free diet for affected children. Many potential predictive factors (e.g. environmental, infectious, birth-related, dietary) have been investigated for their association with CD [15–19]. The evidence for these risk factors is varied [20–22]. Guidelines are often based on symptoms, a couple of associated conditions or predictive factors, such as family history [8–11].

The James Lind Alliance “Top 10” priorities for research on CD highlight the need for finding the risk factors for the development of CD, helping healthcare professionals to achieve earlier diagnosis and to better understand the association between CD and other conditions [23]. These priorities frame the issue addressed by this systematic review which aimed to synthesize the available evidence on risk factors to make this evidence useful in a primary care setting.

### Objectives

In this systematic review, our objectives were to 1) identify factors associated with developing CD from the published literature and 2) to combine data across studies to determine the pooled effect-size of the risk for most frequently reported factors.

## Methods

The reporting for this review was based on the Preferred Reporting Items for Systematic Reviews (PRISMA) reporting guidelines [24]. The protocol was registered prior to commencing on the International Prospective Register of Systematic Reviews (PROSPERO), ID: CRD42022330862 [25]. Amendments to the protocol were to narrow the focus to coeliac disease and to expand the predictive factors included in meta-analysis to all those reported in ≥5 studies.

### Data sources & searches

Literature was searched from MEDLINE(OvidSP), EMBASE(OvidSP), Cochrane Database of Systematic Reviews and Cochrane Central Register of Controlled Trials (Cochrane Library, Wiley) from inception (1966 for MEDLINE, 1947 for EMBASE) to April 9, 2024. The search strategy included the broad key terms for the conditions of interest, for children and young people and for risk factors (Table A1 in S1 File). The initial search in May 2022 included keywords for 3 chronic conditions, the update in April 2023 and April 2024 focused on Coeliac Disease. No date or language limits were applied. A forward and backward citation search was conducted on included studies and relevant review articles using citationchaser [26]. All references were exported to Covidence for deduplication [27].

### Study selection

Studies were selected for inclusion if they:

- Were observational studies or randomized trials.
- Reported an effect measure/an outcome from a statistical test for a risk/predictive factor for CD (see definition below).
- Had a comparison group without the factor of interest.
- Included data on children/young people aged 0–25 years.
- Were published in any country, language or years of publication.
- Reported on a predictive factor relevant to/possible to assess in a primary care context.

Studies were excluded if they:

- Reported risk/predictive factors not relevant to primary care (such as genetic factors).
- Focused on signs/symptoms only.
- Were case reports, case series and protocols.
- Were review articles; however, if they were topically relevant, they were tagged, and their references screened for inclusion.

Titles/abstracts and full texts were independently assessed by two reviewers for eligibility (CF and either AP or DS). Disagreements were resolved by discussion. At the stage of full text review, the specific reason for exclusion was documented.

### Data collection & synthesis

Data were extracted from each report by CF and checked by a second reviewer (AP or DS). For studies with missing data, authors were contacted if the article was published within the past ten years.

For each report, data extracted included:

- The country/countries in which the study was conducted.
- The population size (total number of participants and number with CD).
- The eligible risk factor(s) investigated in the study.
- How each risk factor was defined/measured/categorized.
- The effect measure for each risk factor: odds ratio (OR), hazard ratio (HR), relative risk (RR), p-value if comparing proportions etc.
- Number of participants with and without risk factor in CD and non-CD group if needing to calculate an OR.
- Means and standard error of the mean (SEM) in CD and non-CD group (only applicable for height and weight).

Adjusted effect measures were selected over unadjusted if both were available. If a ratio estimate was not provided, 2x2 tables were constructed when possible and unadjusted ORs and 95% confidence intervals calculated.

Height and weight are continuous variables which cannot be appropriately compared through a ratio. For these variables, means and standard errors of the means for the CD and non-CD groups were extracted for use in calculating the standardized mean difference (SMD).

For study groups with multiple reports/published studies using data on the same study population, data were extracted for each report and then analysed together as a study group. If multiple reports included the same risk factor, one was selected to represent the study group. This choice was made hierarchically based on:

1. The availability of an adjusted effect measure.
2. The largest sample size of participants included in the analysis.
3. The consistency of the definition of the factor with other studies reporting on the same factor.
4. The recency of the publication.

CD was defined differently across studies depending on the year of publication and the availability of different types of testing. If only coeliac autoimmunity was assessed, this was used as an acceptable endpoint given the recent shift to diagnosis based on serology alone and not always requiring a biopsy in some jurisdictions. If data were presented on both coeliac autoimmunity and confirmed cases of CD for the same factor within a study, we extracted data based on the more specific definition of confirmed CD.

Table 1 provides the definitions used when extracting data for the factors included in meta-analysis. Extracted effect measures were rescaled to match these definitions to allow data to be combined across studies when necessary.

**Table 1:** Definitions used when extracting data on each predictive factor for meta-analysis.

#	Factor	Definition
**1**	Sex (Female)	Female vs male.
**2**	Breastfeeding	Any breastfeeding compared to none or little. Variable definitions on duration (such as >30 days, >4 months, >6 months or per month continuous) but all comparing more to less.
**3**	C-Section	C-section vs vaginal delivery.
**4**	Height (Taller)	Greater height at variable age (either fixed age point in early childhood or relative to CD diagnosis) presented as z-score or mean height.
**5**	Gluten Introduction (Later)	Later age of Gluten introduction relative to earlier, typically >6 months relative to between 3 and 6 months of age.
**6**	Weight (Heavier)	Greater weight at variable age (either fixed age point in early childhood or relative to CD diagnosis) presented as z-score or mean weight.
**7**	First Degree Relative with CD	Has a first degree relative with CD (some studies may specify specifically mother, sibling etc but would be included in this category).
**8**	Infections (More in Early Life)	Any infection or more frequent infections in early childhood (infancy/first year of life, first 18 months, at time of gluten introduction etc.)
**9**	Maternal Education (Higher)	High maternal education compared to low. Usually university/some level greater than high school compared to high school or less.
**10**	Birth Season (Summer)	Born in summer (defined as May/June to Aug/Sept) relative to winter or to the rest of the year.
**11**	Respiratory Infections (More in Early Life)	Presence or greater frequency of respiratory infections in early childhood, first 6 months, first year of life, second year of life. Some investigated a specific virus/presentation (i.e. RSV, viral croup) whereas others reported respiratory infections more broadly.
**12**	Antibiotic Use (Early in Life)	Any antibiotic use in early childhood, infancy, or first year of life.
**13**	Gastroenteritis (More in Early Life)	Presence or greater frequency of gastroenteritis in early childhood, first year of life, second year of life. Some investigated a specific virus (i.e. rotavirus, enterovirus) whereas others reported gastroenteritis more broadly.
**14**	Breastfeeding at Gluten Introduction	Breastfeeding at the time of gluten introduction (some studies only looked at time point of first gluten introduction, some at continuation for a period after).
**15**	Gluten Ingestion (More)	Greater amount of gluten ingestion (in grams/day, number of gluten rich meals etc) compared to less at variable age in early childhood such as 2 weeks after gluten introduction or at 18 months of age.
**16**	BMI (Higher)	Higher BMI (usually overweight) compared to normal/underweight. One study compared normal/overweight to underweight.
**17**	Birth Weight (Normal/High)	Normal/high birth weight (typically >2500g or >3000g) compared to low birth weight.
**18**	Age at T1DM Diagnosis (Older)	Older age compared to younger age. Typically, younger reference group <5 years.
**19**	Gestational Age (Premature)	Premature gestational age (typically <36 or 37 weeks) compared to full term.
**20**	Race	White/western compared to Hispanic, non-western, non-white etc. (as defined within the individual studies).
**21**	Maternal Age (Older)	Older maternal age, typically >30 compared to <30 or <25.
**22**	Thyroid Disease	Any autoimmune thyroid disease (hypothyroidism, thyroiditis, autoantibody positive etc).
**23**	Income (High)	High or high-medium income family/parental income compared to low.
**24**	Maternal Smoking (During Pregnancy)	Maternal smoking at any point during pregnancy compared to not.
**25**	Older Siblings	Child has one or more older sibling compared to no older siblings.
**26**	Vitamin D	Taking supplements vs not, or high/normal levels compared to deficient at different age/time points in early childhood.
**27**	Parity (Multi-parity)	Mom was multiparous (had a previous pregnancy) at time of delivery of child being investigated for CD.
**28**	T1DM	Child has a diagnosis of Type 1 Diabetes Mellitus (T1DM)

### Risk of bias assessment

The methodological quality and risk of bias of individual studies were assessed using the Newcastle-Ottawa Scale for cohort studies and for case-control studies [28]. A modified version of the Newcastle-Ottawa Scale was used for cross-sectional studies, and is included in the supplemental materials (Document A1 in S1 File) [29]. The scores were converted to Agency for Healthcare Research and Quality standards of good, fair or poor quality. Randomized trials were assessed using the Risk of Bias 2 (RoB 2) tool [30].

### Eligibility for meta-analysis

Meta-analysis was conducted for factors reported in ≥ 5 studies. Age was excluded from meta-analysis as the way age was grouped and reported was inconsistent across studies so there were not at least three studies using similar definitions to be combined. Dietary Composition was similarly excluded as each study investigated different aspects of diet and dietary composition and could not be combined. Birth year was similarly excluded because studies investigated different years and could not be combined. T1DM was selected for meta-analysis post-hoc despite being reported in only four studies for face validity given its clinical relevance, inclusion in guidelines and known association with coeliac disease [8–11].

### Meta-analyses

We calculated pooled OR estimates using log OR and their 95% confidence intervals (CIs) or standardized mean difference (SMD) for each factor with a random-effects meta-analysis using the Hartung-Knapp-Sidik-Jonkman (HKSJ) method [31]. Standardized mean difference was used for height and weight because the range of how these variables were reported across included studies (z-scores, percentiles and actual units of height and weight). Given the range of ratio effect measures reported in the included studies, ORs, HRs and RRs were all combined in the initial meta-analyses [32, 33].

Sensitivity analyses were conducted restricting to 1) only high or fair quality studies based on risk of bias assessment, 2) studies with >100 participants, 3) studies reporting odds ratios (as opposed to hazard ratios or relative risks) and 4) studies reporting adjusted effect measures. Sensitivity analysis was only performed if at least 3 studies could be combined for that factor. A sensitivity analysis was also conducted comparing the HKSJ random effects model to the DerSimonian and Laird (DL) random effects model. Statistical significance for all meta-analyses was assessed at a threshold of p-value under 0.05.

Reporting bias was assessed through funnel plot asymmetry. For height and weight (assessed through SMD and not pooled OR), Egger’s test was used to test for funnel plot asymmetry.

Given the nature of this review being predominantly observational trials, we elected not to use the Grading of Recommendations Assessment, Development, and Evaluation (GRADE) approach to assess certainty of evidence. Observational studies can provide the optimum evidence for the predictive value of an indicator, yet, in GRADE, observational studies start with a low level of quality [34, 35]. The comparison of the HKSJ and DL random effects models and sensitivity analyses restricting to adjusted measures only and excluding high risk of bias also contribute to an assessment of the certainty of evidence.

All analyses were conducted using STATA version 18 (Standard Edition, StataCorp, College Station, TX).

### Patient & public involvement

We sought input from individuals with a range of experiences related to CD as parents and patients. Consultation took place at the stage of planning and drafting the protocol and throughout the analysis. The input from our patient and public partners was considered in study design and in plans for disseminating the study results. Speaking to parents and patients in the design stage informed the research question about the types of predictive factors focused on in this review and the decision to exclude symptoms which are already well understood and considered in the diagnostic process. Input from patients and parents at the analysis and write-up stage informed the clinical implications section of the discussion.

## Results

### Study selection & characteristics

**Fig 1:**
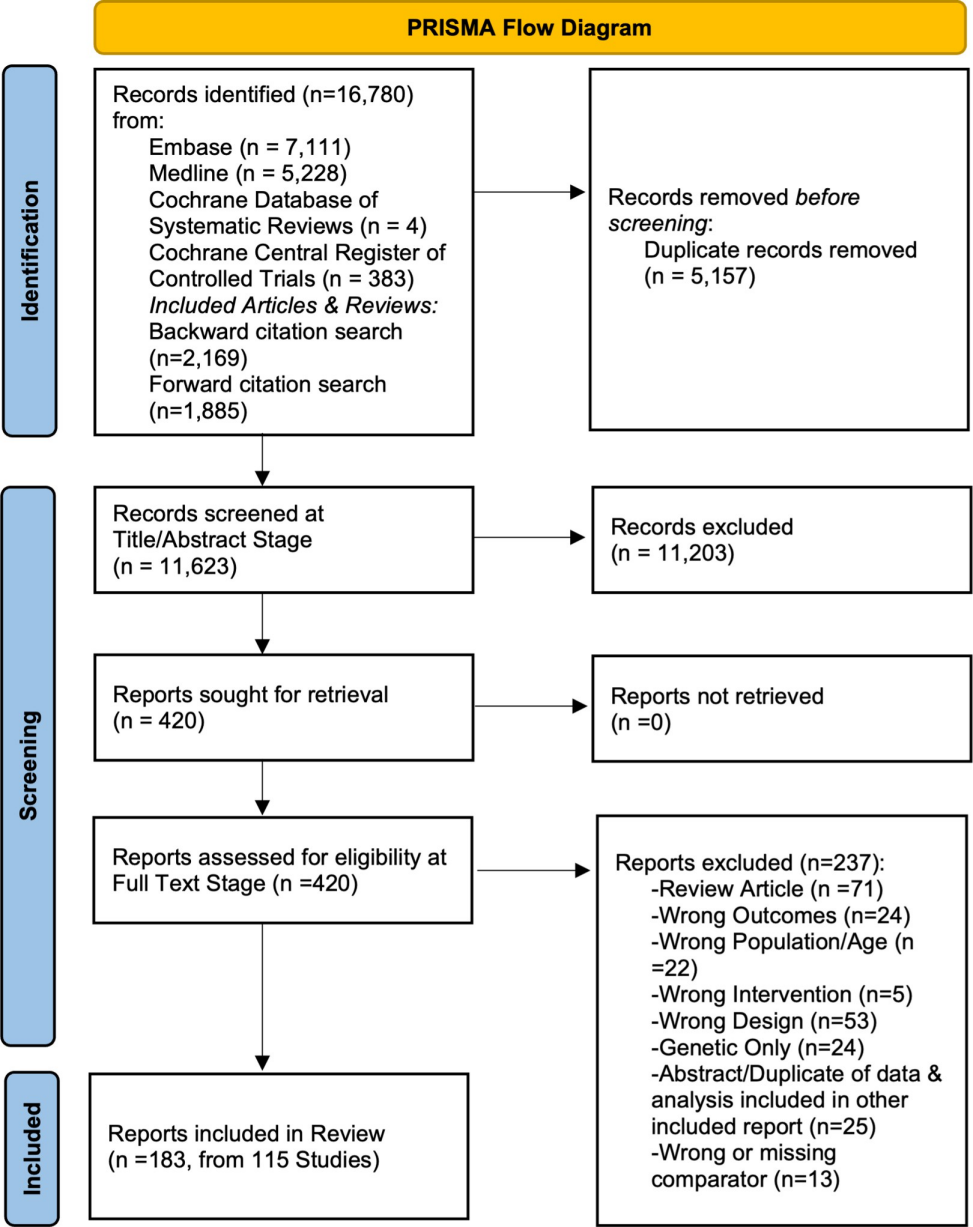
Flowchart for eligible studies identification according to PRISMA 2020 guidelines. PRISMA (Preferred Reporting Items for Systematic Reviews and Meta-Analyses).

Our database searches and the forward/backward citation search of all included records and relevant reviews identified 11575 records after de-duplication. We selected 410 records for full text review, of which 183 fulfilled inclusion criteria (see Fig 1). Included articles were published from 1983–2024.

The summary characteristics of all included studies (by study group), including the predictive factors reported in each, are presented in full in Table 2, and the references are included in Table A2 in S1 File. Of the 183 reports, 8 were randomized trials, 48 were case-control studies, 119 were cohort studies and 8 were cross-sectional. 102 reports were unique studies and the remainder presented data from 13 study groups which were analysed together, giving a total of 115 studies. Reports presented data from a range of countries (number of reports given in parentheses): Sweden (43), International cohorts (23), Italy (21), USA (15), Norway (13), Israel (12), Denmark (7), UK (7), Finland (6), Netherlands (4), Spain (5), Turkey (5), Germany (4), Iran (2), Jordan (2), Switzerland (2), Saudi Arabia (2), Cyprus (1), France (1), Greece (1), UAE (1), India (1), Poland (1), Oman (1), Brazil (1), Ethiopia (1), and Pakistan (1).

**Table 2:** Characteristics of eligible studies.

Extracted Study ID (Author Year)	Country	Study Type	Total Included	With CD	# of Factors	Predictive Factors
**Ahmed 2022**	UAE	Cohort, Abstract Only	898	93	3	First Degree Relative with CD, Age at T1DM Diagnosis, Thyroid Disease
**Al-Hussaini 2012**	Saudi Arabia	Cohort	106	12	5	Age at T1DM Diagnosis, BMI, Sex, Height, Weight
**Al-Sinani 2013**	Oman	Cross-sectional	93	5	2	Age at T1DM Diagnosis, Sex
**Ascher 1997**	Sweden	Case-Control	81	8	4	Breast Feeding, Milk Introduction, Breast Feeding at Gluten Introduction, Gluten Ingestion
**Assa 2018**	Israel	Cohort	2,001,353	10,566	1	Season of Birth
**Auricchio 1983**	Italy	Case-Control	505	216	2	Gluten Introduction, Breast Feeding
**Auricchio 2021**	Italy	Cohort, Abstract Only	373	27	1	Gluten Ingestion
**Batista 2012**	Brazil	Cross-sectional	2,181	147	1	Autism Spectrum Disorder
**Beser 2019**	Cyprus	Cohort	3,792	15	10	Maternal Education, Income, Breast Feeding, Gluten Introduction, BMI, Height, Weight, Paternal Education, Sex, Thalassemia in the Family
**Beyerlein 2017**	Germany	Cohort	294,138	853	2	Gastroenteritis, Respiratory Infections
**Bingley 2004**	UK	Cohort	5,470	54	2	Height, Weight
**Bittker 2019**	USA	Case-Control	573	332	8	Ear Infection, Antibiotics, Breast Feeding, Vitamin D, Quantity of Cows Milk, Type of Water, Type of Cows Milk, Gluten Introduction
**Boechler 2023**	USA	Cohort	968,524	1,704	6	Sex, C-section, Gestational Age, Antibiotics, PPI use, H2RA use
**Calderoni 2016**	Italy	Cross-sectional, Cohort	382	10	1	Autism Spectrum Disorder
**Capriati 2015**	Italy	Cohort	439,990	596	1	Season of Birth
**Carlsson 2002**	Sweden	Case-Control	403	76	1	Enterovirus during pregnancy
**Castellaneta 2015**	Italy	Cohort	446	38	7	T1DM, Sex, Age at T1DM Diagnosis, C-section, Breast Feeding, Infections, Gastroenteritis
**Cerutti 2004**	Italy	Cohort	4,322	292	3	Sex, Thyroid Disease, Age at T1DM Diagnosis
**Cilleruelo 2016**	Spain	Cohort	1,291	15	5	Breast Feeding, Breast Feeding at Gluten Introduction, C-section, First Degree Relative with CD, Sex
**Dalgic 2011**	Turkey	Cohort	20,190	95	7	Income, Maternal Education, Paternal Education, Breast Feeding, First Degree Relative with CD, Gluten Introduction, Sex
**DydensborgSander 2018**	International	Cohort	1,588,485	3,314	1	C-section
**DydensborgSander 2019**	International	Cohort	1,706,113	3,346	1	Antibiotics
**Fälth-Magnusson 1996**	Sweden	Case-Control	360	72	3	Breast Feeding at Gluten Introduction, Gluten Introduction, Mode of Gluten
**Francavilla 2011**	Italy	Case-Control, Abstract Only	14,500	2,130	4	Breast Feeding, C-section, Infections, Sex
**Gatti 2019**	Italy	Cohort	4,265	43	6	Sex, Atopy, First Degree Relative with CD, Weight, Height, BMI
**Gaylord 2020**	USA	Cohort	88	30	1	Organic Pollutant Exposure
**Giannotti 2001**	Italy	Cross-sectional	17,264	100	1	Williams Syndrome
**Greco 1988**	Italy	Case-Control	2,150	201	2	Breast Feeding, Gluten Introduction
**Greco 1990**	Italy	Case-Control	262	82	2	Asthma, Atopy
**Gudeta 2022**	Ethiopia	Cohort	1,046	12	8	Sex, Gluten Ingestion, H. Pylori, Maternal Latent TB, Older Siblings, Breast Feeding, Height, Weight
**Güngör 2013**	Turkey	Case-Control	752	1	1	ADHD
**Hansen 2007**	Denmark	Case-Control	212	11	6	Sex, Age at T1DM Diagnosis, Height, Weight, BMI, Thyroid Disease
**Hemming-Harlo 2019**	Finland	Randomised Trial	5,764	48	1	Rotavirus Vaccination
**Hyytinen 2017**	Finland	Randomised Trial	230	10	1	Hydrolysed Formula
**Inns 2021**	UK	Cohort	880,629	1,657	6	Rotavirus Vaccination, Deprivation, Sex, GP Consultations, UK Region, Birth Year
**Isikay 2014**	Turkey	Case-Control	818	0	1	Breath Holding Spells
**Ivarsson 2013**	Sweden	Cross-sectional	208,499	899	2	Sex, Breast feeding at Gluten Introduction
**Ji 2011**	Sweden	Cohort	1,050,569	1,192	1	Parental Country of Birth
**Kahrs 2019**	Norway	Case-Control	74	25	1	Gastroenteritis
**Kakleas 2010**	Greece	Cohort	105	9	2	Age at T1DM Diagnosis, BMI
**Kårhus 2017**	Norway	Cohort	872,459	3,415	1	Respiratory Infections
**Kalvandi 2021**	Iran	Cohort	120	14	4	Sex, Breast Feeding, City, Maternal Education
**KeceliBasaran 2021**	Turkey	Case-Control	210	4	5	Sex, Breast Feeding, Height, Weight, Vitamin D
**Kori 2022**	Israel	Cohort	90	12	1	Sex
**Kuja-Halkola 2017**	Sweden	Cohort	146,161	669	1	Birth Weight
**Lebwohl 2012**	Sweden	Case-Control	67,695	11,308	1	Season of Birth
**Lebwohl 2013**	USA	Cross-sectional	6,169	160	1	H. Pylori
**Lebwohl 2014**	Sweden	Case-Control	7,293	1,218	1	PPI use
**Lehtinen 2016**	Finland	Randomised Trial	32,176	17	1	HPV Vaccination
**Leonard 2023**	International	Cohort, Abstract only	423	54	5	Antibiotics, PPI Use, Probiotics, Infections, Sex
**Lewy 2009**	Israel	Cohort	1,040,558	431	1	Season of Birth
**Lionetti 2014**	Italy	Randomised Trial	553	66	3	Breast Feeding, Breast Feeding at Gluten Introduction, Gluten Introduction
**Lionetti 2017**	Italy	Cohort	431	71	1	C-section
**Lionetti 2020**	Italy	Case-Control	262	131	1	Vitamin D
**Ludvigsson 2013**	Sweden	Case-Control	70,654	11,802	1	Intussusception
**Ludvigsson 2013_1**	Sweden	Case-Control	65,285	10,904	1	Autism Spectrum Disorder
**Ludvigsson 2013_2**	Sweden	Case-Control	70,654	11,802	1	Head Trauma
**Lurz 2008**	Switzerland	Cohort	206	94	4	Sex, Age, Height, Weight
**Maleki 2023**	Iran	Case-Control	372	186	12	Age, Birth Weight, City, C-section, Dietary Composition, BMI, Sex, Maternal Education, Income, Gestational Age, Breast Feeding, Family History Autoimmune
**Mårild 2011**	Sweden	Case-Control	65,636	11,749	6	Small for Gestational Age, Birth Weight, Gestational Age, APGAR, Infections, C-section
**Marild 2013**	Sweden	Case-Control	33,933	2,933	1	Antibiotics
**Mårild 2013_1**	Sweden	Case-Control	65,636	11,749	1	Trisomy 21/Down syndrome
**Mårild 2016**	Sweden	Case-Control	42,040	7,548	1	Turner Syndrome
**Miranda 2017**	France	Cohort	2,252,716	188	1	HPV Vaccination
**Moos 2020**	Denmark	Cohort	206,900	347	3	Sex, Vitamin D, Season of Birth
**Moyer 2018**	USA	Cohort, Abstract Only	961	52	4	Age, Race, Sex, Thyroid disease
**Naddei 2022**	Italy	Cohort	329	8	3	Sex, Age at JIA Diagnosis, Family History Autoimmune
**Namatovu 2014_2**	Sweden	Cohort	2,080	2,080	1	Neighbourhood
**Narang 2017**	India	Case-Control	646	324	4	H. Pylori, Age, Sex, Height
**Norström 2020**	Sweden	Cohort	11,029	319	2	Maternal Occupation, Maternal Education
**Nusier 2010**	Jordan	Cohort	1985	16	3	Height, Weight, BMI
**Odeh 2019**	Jordan	Cohort	538	49	3	Age at T1DM Diagnosis, Thyroid Disease, Sex
**Oikarinen 2021**	Finland	Case-Control	94	41	1	Respiratory Infections
**Patel 2018**	USA	Case-Control	282	94	10	Asthma, BMI, Race, Atopy, First Degree Relative with CD, Family History Asthma, Family History Atopy, Flu Vaccination, T1DM, Thyroid Disease
**Pelayo 2020**	Spain	Case-Control	186	93	6	Infections, Respiratory Infections, Gastroenteritis, Ear Infection, UTI, Antibiotics
**Peters 2001**	Germany	Case-Control	280	143	3	Breast Feeding, Gluten Introduction, Breast Feeding at Gluten Introduction
**Prosperi 2021**	Italy	Cohort	362	9	1	Autism Spectrum Disorder
**Renata 2022**	Italy	Case-Control	83	27	2	Gluten Ingestion, Dietary Composition
**Roberts 2009**	UK	Cohort	248,521	90	19	Breast Feeding, APGAR, Birth Weight, Breech, C-section, Deprivation, Forceps, Gestational Age, Maternal Marital Status, Maternal Age, Maternal Blood Group, First Degree Relative with CD, Maternal Rhesus Status, Maternal Smoking, Multiples, Parity, Pre-eclampsia, Sex, Birth Year
**Roman 2010**	Spain	Case-Control, Abstract Only	1,737	993	4	First Degree Relative with CD, Gastroenteritis, Respiratory Infections, Breast Feeding at Gluten Introduction
**Rutz 2002**	Switzerland	Cohort	1,450	8	3	Height, Weight, Sex
**Saadah 2012**	Saudi Arabia	Cohort	430	48	4	Age at T1DM Diagnosis, Height, Weight, Thyroid Disease
**Saari 2015**	Finland	Cohort	51,509	177	2	Height, BMI
**Sadiq 2022**	Pakistan	Cross-sectional	1,090	112	3	Sex, Age, Gastroenteritis
**Savilahti 2018**	Finland	Randomised Trial	867	11	7	Probiotics, Breast Feeding, Respiratory Infections, Antibiotics, Atopy, T1DM, C-section
**Schaub 2015**	USA	Cohort, Abstract Only	758	15	1	Race
**Segerstad 2018**	Sweden	Case-Control	828	207	1	Milk Powder Intake
**Selimoğlu 2013**	Turkey	Case-Control	168	84	1	Craniofacial Features
**Simre 2016**	International	Cohort	3,310	29	4	Country, Breast Feeding, Gluten Introduction, Infections
**Simre 2019**	International	Case-Control	58	29	3	Infections, Gastroenteritis, Respiratory Infections
**Stagi 2005**	Italy	Case-Control	309	11	1	Juvenile Idiopathic Arthritis
**Stahl 2020_1**	USA	Cohort	9,973	149	4	Sex, First Degree Relative with CD, Race, Age
**Stene 2006**	USA	Case-Control	162	54	8	Sex, Race, Season of Birth, Maternal Education, Breast Feeding, Age Starting Daycare, Older Siblings, Gastroenteritis
**Tanpowpong 2013**	USA	Cohort	1,964	1,964	1	Season of Birth
**Tanpowpong 2023**	USA	Cohort	44,539	173	11	Sex, Birth Weight, Multiples, Gestational Age, Maternal Age, Race, Gestational Hypertension, Maternal Diabetes, C-section, Maternal BMI, Gestational Weight Gain
**Tapia 2021**	Norway	Case-Control	220	25	1	Infections
**Tjernberg 2014**	Sweden	Case-Control	22,937	3,835	1	Respiratory Infections
**vanderPals 2014**	Sweden	Cohort	12,466	239	3	Height, Weight, BMI
**Walkowiak 2010**	Poland	Case-Control	3,517	16	1	Cystic Fibrosis
**Whyte 2013**	UK	Cross-sectional	298,530	232	1	Deprivation
**Zacay 2024**	Israel	Cohort	14,232	2,372	1	Fractures
**Study Group: Soroka University Medical Center**						
**Bendersky 2020**	Israel	Cohort, Abstract Only	7,337	40	1	C-section, Season of Birth, Maternal Smoking, First Degree Relative with CD, Small for Gestational Age, Maternal GBS
**Daniel 2019**	Israel	Cohort	308,903	699	1	
**Davidesko 2021**	Israel	Cohort	139,232	500	1	
**Gutvirtz 2024**	Israel	Cohort, Abstract Only	356,109	1,960	1	
**Karur 2021**	Israel	Cohort	241,319	940	1	
**Pariente 2019**	Israel	Cohort, Abstract Only	242,342	973	1	
**Steiner 2019**	Israel	Cohort	225,600	856	1	
**Yoles 2018**	Israel	Cohort, Abstract Only	195,457	732	1	
**Study Group: The Environmental Determinants of the Diabetes in the Young (TEDDY)**						
**Aronsson 2015**	International	Cohort	6,436	307	9	Country, Maternal Smoking, Maternal Education, Maternal Age, Breast Feeding When Introducing Gluten, First Degree Relative with CD, Gluten Introduction, Season of Birth, Sex, Breastfeeding, Energy Intake, Gluten Ingestion, Vitamin D, First-degree Relative with T1DM, C-section, Age Starting Daycare, Older Siblings, Antibiotics, Maternal BMI, Gestational Weight Gain, Maternal Diabetes, Birth Height, Gestational Age, Hospital Admission for Neonatal Infection, Gastroenteritis, Maternal Gluten Intake, Probiotics, Dietary Composition
**Aronsson 2015_1**	Sweden	Case-Control	2,208	146	4	
**Aronsson 2019**	International	Cohort	5,641	447	1	
**Aronsson 2021**	International	Case-Control	924	281	1	
**Hagopian 2017**	International	Cohort	5,891	341	4	
**HårdAfSegerstad 2022**	Sweden	Cohort	2,088	242	1	
**HårdAfSegerstad 2023**	International	Cohort	6,677	529	5	
**Kemppainen 2017**	International	Cohort	6,327	283	11	
**Kemppainen 2017_1**	International	Cohort	6,558	783	1	
**Koletzko 2018**	International	Cohort	6,087	343	15	
**Laitinen 2018**	International	Cohort	6,312	435	1	
**Lindfors 2019**	International	Case-Control	166	83	1	
**Liu 2014**	International	Cohort	6,034	312	3	
**Uusitalo 2015**	International	Cohort	6,546	359	5	
**Uusitalo 2019**	International	Cohort	6,520	455	1	
**Yang 2017**	International	Cohort	6,627	409	3	
**Study Group: Swedish Medical Birth Registry**						
**Adlercreutz 2015**	Sweden	Cohort	768,395	3,817	15	Season of Birth, Birth Year, C-section, Maternal Age, Maternal Country of Birth, Maternal Education, Income, Parity, Maternal Smoking, Social Allowance, Gestational Age, Sex, Small for Gestational Age, Malformation, T1DM, BMI, Premature Rupture of Membranes, Birth Weight, APGAR, Maternal Infections, Maternal-Child Blood Group Incompatibility, Infections, Neonatal Jaundice, Pre-eclampsia, Older Siblings, Sweden Region, Maternal Diabetes, Maternal Hypertension, Maternal Kidney Disease, Maternal UTI
**Namatovu 2014**	Sweden	Cohort, Abstract Only	1,912,204	6,596	3	
**Namatovu 2016**	Sweden	Cohort	1,912,204	6,569	3	
**Namatovu 2016_2**	Sweden	Cohort	1,912,204	6,569	11	
**Sandberg-Bennich 2002**	Sweden	Cohort	1,182,056	3,482	10	
**Wingren 2011**	Sweden	Cohort	792,401	2,264	10	
**Wingren 2012**	Sweden	Cohort	681,954	2,635	10	
**Wingren 2012_1**	Sweden	Cohort	789,508	2,893	1	
**Wingren 2012_2**	Sweden	Cohort	792,401	2,264	4	
**Wingren 2012_3**	Sweden	Cohort	792,401	2,907	10	
**Study Group: BabyDiab**						
**Hummel 2007**	Germany	Cohort	1,511	16	6	Birth Weight, Breast Feeding, Infant food Supplementation, Gluten Introduction, First Degree Relative with CD, Sex
**Winkler 2019**	Germany	Cohort	2,441	117	1	
**Study Group: Norwegian Mother and Child Cohort Study (MoBa)**						
**Emilsson 2015_1**	Norway	Cohort	108,478	650	15	Birth Weight, Breech, Birth Height, Child Growth Indication for C-Section, C-section, Gestational Age, Gestational Hypertension, Maternal Diabetes, Maternal Education, Maternal Indication for C-Section, Maternal Smoking, Parity, Paternal smoking, Pre-eclampsia, Small for Gestational Age, Height, Weight, Gluten Ingestion, Gluten Introduction, Maternal Fibre Intake, Maternal Gluten Intake, Infections, Gastroenteritis, Respiratory Infections, Maternal Infections, Maternal Antibiotics, Maternal Paracetamol, Vitamin D, Maternal vitamin D, Maternal Iron Supplementation, Child Iron Supplementation, Breast Feeding, Breast feeding at Gluten Introduction
**Kahrs 2017**	Norway	Cohort	58,674	440	2	
**Kahrs 2018**	Norway	Case-Control	67,608	738	2	
**Lund-Blix 2019**	Norway	Cohort	67,608	738	2	
**Lund-Blix 2020**	Norway	Cohort	85,898	927	2	
**Mårild 2015**	Norway	Cohort	72,921	581	3	
**Marild 2017_1**	Norway	Cohort	84,274	617	4	
**Marild 2017_2**	Norway	Case-Control	986	416	2	
**Mårild 2019_2**	Norway	Cohort	536,861	1,919	1	
**Størdal 2013**	Norway	Cohort	78,846	314	2	
**Størdal 2013_2**	Norway	Cohort	82,167	324	3	
**Study Group: The Health Improvement Network (THIN)**						
**Tata 2015**	UK	Cohort, Abstract Only	798,343	708	1	First Degree Relative with CD, Adrenal Insufficiency, Age at T1DM Diagnosis, Sex, Year of T1DM Diagnosis, Country, Deprivation, Birth Year
**Vajravelu 2018**	UK	Cohort	9,180	196	4	
**Zingone 2015**	UK	Cohort	2,063,421	1,247	4	
**Study Group: PreventCD**						
**Auricchio 2017**	Italy	Cohort	248	31	2	Respiratory Infections, Gastroenteritis, Height, Weight, Country, Gluten Ingestion, Gluten Introduction, Sex, City, Maternal Gluten Intake, First Degree Relative with CD,C-section, Sex
**Auricchio 2020**	International	Cohort	944	113	2	
**Crespo-Escobar 2017**	International	Cohort, Randomised Trial	944	95	4	
**CrespoEscobar 2018**	Spain	Randomised Trial	225	26	4	
**Meijer 2022**	International	Cohort	944	135	6	
**Vriezinga 2014**	International	Cohort, Randomised Trial	963	80	1	
**Study Group: Swedish Celiac Register**						
**Ivarsson 2002**	Sweden	Case-Control	1,881	627	4	Breast Feeding at Gluten Introduction, Gluten Ingestion, Gluten Introduction, Food Given at Gluten Introduction, Age, Birth Year, Sex, Season of Birth, Income, Infections, Older Siblings, MMR Vaccine, HiB Vaccine, BCG Vaccine, Pertussis
**Ivarsson 2003**	Sweden	Cohort	4,408,816	4,302	3	
**Ivarsson 2003_2**	Sweden	Cohort	882,122	2,151	3	
**Myleus 2012_1**	Sweden	Case-Control	954	373	5	
**Myleus 2012_2**	Sweden	Case-Control	1,015	392	6	
**Study Group: Danish Medical Birth Registry**						
**Andersen 2020**	Denmark	Cohort	2,672,708	7,132	1	C-section, First Degree Relative with CD, First Degree Relative with T1DM, First Degree Relative with Rheumatoid Arthritis, Ear Infection, Respiratory Infections, Breast Feeding, Infections, Antibiotics, Dietary Composition, Maternal Thiopurines
**Andersen 2021**	Denmark	Cohort	2,699,449	9,231	3	
**Crawley 2022**	Denmark	Abstract Only, Cohort	1,266	33	6	
**Jølving 2021**	Denmark	Cohort	1,308,221	3,409	1	
**Sevelsted 2015**	Denmark	Cohort	1,900,000	1,944	1	
**Study Group: Friuli-Venezia Giulia Region**						
**Canova 2014**	Italy	Cohort	203,557	1,227	14	Maternal Education, Birth Season, Parity, Birth Weight, Gestational Age, APGAR, Breech, Maternal Age, Antibiotics, Hospitalization for Neonatal Infection, Hospitalization for Neonatal Intestinal Infection, C-section, Older Siblings, Sex, Asthma, Age
**Canova 2015**	Italy	Cohort	143,144	717	4	
**Study Group: Generation R**						
**Barroso 2018**	Netherlands	Cohort	1,997	27	1	Dietary Composition, Breast Feeding, Gluten Introduction, Maternal Education, Income, C-section, Parity, Maternal Smoking, Sex, Race, Birth Weight, Gastroenteritis, Respiratory Infections, Vitamin D, Age Starting Daycare, Maternal Age, Antibiotics, Herpesvirus Infection, Maternal vitamin D, Outdoor Play
**Jansen 2014**	Netherlands	Cohort	1,679	26	14	
**Jansen 2016**	Netherlands	Cohort	4,420	31	3	
**vanderVelde 2023**	Netherlands	Cohort	3,994	54	12	
**Study Group: All Babies in Southeast Sweden (ABIS)**						
**Ludvigsson 2005**	Sweden	Cohort	15,397	53	2	Maternal Smoking, Maternal BMI, Sex, Crowded Living, Older Siblings, Maternal Marital Status, Maternal Alcohol, Maternal Country of Birth, Maternal Education, Maternal Living Condition, Paternal Age, Paternal Country of Birth, Paternal Education, Paternal Living Condition, Paternal Employment, Maternal Employment, Serious Life Event, Parenting Stress, Parental Worries, Psychological Stress, Maternal Antibiotics, Breast feeding, Gastroenteritis, Gluten Introduction, Infections, Income
**Ludvigsson 2007**	Sweden	Cohort	15,867	45	14	
**Mårild 2010**	Sweden	Cohort	8,805	83	4	
**Mårild 2014**	Sweden	Cohort	8,729	46	1	
**Welander 2010**	Sweden	Cohort	9,408	44	6	
**White 2023**	Sweden	Cohort	17,055	228	1	
**Study Group: Diabetes Autoimmunity Study in the Young (DAISY)**						
**Liu 2017**	USA	Cohort	1,339	66	4	Race, First-degree relative with T1DM, First Degree Relative with CD, Sex, Gluten Ingestion, Gluten Introduction, Milk Introduction, Oats Introduction, Rice, Birth Weight, Breast Feeding at Gluten Introduction, Breast Feeding, Maternal Age, Maternal Education, BMI, Height, Weight
**Marild 2018**	USA	Cohort	1,875	85	1	
**Norris 2005**	USA	Cohort	1,560	51	14	
**Stahl 2020_2**	USA	Cohort	1,979	71	3	

We identified 145 predictive factors, of which 30 were reported in ≥5 studies. Excluding age, dietary composition and birth year and adding T1DM (as discussed in the methods), 28 factors were included in the meta-analysis. The frequency with which each predictive factor was reported by a unique study/study group is presented in Table 3 for those reported in ≥5 studies and in Table A3a/A3b in the S1 File for those reported in ≤4 studies. Table A4 in the S1 File summarizes which studies reported a predictive factor as significant and which as non-significant for all risk factors reported in ≥5 studies. Age was reported in seven studies and the extracted data is presented in Table A5 in S1 File given its potential relevance, but given the heterogeneity of definitions in the individual studies, it was not possible to include age in the meta-analysis.

**Table 3:** Predictive factors for a diagnosis of coeliac disease, and the frequency in which they were reported in the included studies. This table is restricted to factors reported in ≥5 studies and therefore eligible for inclusion in the meta-analysis. Some studies did not report sufficient data to allow for inclusion in meta-analysis, so the total studies figures included in this table maybe higher than the number of included studies for the corresponding meta-analysis.

#	Factor	Total Studies	Significant
**1**	Sex	41	21
**2**	Breastfeeding	26	6
**3**	C-Section	18	2
**4**	Height	17	9
**5**	Gluten Introduction	17	3
**6**	Weight	15	7
**7**	First Degree Relative with CD	14	11
**8**	Infections	14	6
**9**	Maternal Education	13	3
**10**	Birth Season	12	8
**11**	Respiratory Infections	12	8
**12**	Antibiotics	12	6
**13**	Gastroenteritis	12	5
**14**	Breastfeeding at Gluten Introduction	10	6
**15**	Gluten Ingestion	11	5
**16**	BMI	11	3
**17**	Birth Weight	11	2
**18**	Age at T1DM Diagnosis	10	6
**19**	Premature Gestational Age	10	1
**20**	Race	9	7
**21**	Maternal Age	8	3
**22**	Thyroid Disease	7	4
**23**	Income	7	3
**24**	Maternal Smoking	7	2
**25**	Older Siblings	7	1
**26**	Vitamin D	6	1
**27**	Parity	5	2
**28**	T1DM	4	4
**Excluded from meta-analysis:**			
**29**	Age	7	5
**30**	Dietary Composition	5	3
**31**	Birth Year	5	2

We excluded studies which did not include a comparator group, which meant we were not able to include studies which suggested potential associations. For example, this led to the exclusion of studies reporting high rates of CD amongst children with Trisomy 21 or T1DM due to a lack of a comparison group without the condition [36, 37].

### Risk of bias

The study quality/risk of bias results are summarized in Table 4 and the score breakdown and justification is available in the S2 File. Most studies were rated as good quality, with only eight of fair quality or having some risk of bias, and one study considered poor quality.

**Table 4:** Study quality and risk of bias assessment for all included studies, assessed using the Newcastle Ottawa Scale (NOS) for cohort studies or Case-Control (C-C) studies, the modified NOS for Cross-Sectional (C-S) studies, or the Risk of Bias 2 (RoB 2) Tool for Randomized Trials.

Study ID	NOS Cohort	Study Quality	Study ID	NOS Cohort	Study Quality	Study ID	NOS C-C Score	Study Quality
Adlercreutz 2015	9	Good	Liu 2017	8	Good	Aronsson 2015_1	9	Good
Ahmed 2022	8	Good	Ludvigsson 2005	8	Good	Aronsson 2021	9	Good
Al-Hussaini 2012	6	Good	Ludvigsson 2007	8	Good	Ascher 1997	8	Good
Andersen 2020	8	Good	Lund-Blix 2019	9	Good	Auricchio 1983	7	Good
Anderson 2021	9	Good	Lund-Blix 2020	9	Good	Bittker 2019	6	Fair
Aronsson 2015	9	Good	Lurz 2008	8	Good	Carlsson 2002	7	Good
Aronsson 2019	9	Good	Mårild 2010	9	Good	Fälth-Magnusson 1996	8	Good
Assa 2018	8	Good	Mårild 2014	9	Good	Francavilla 2011	8	Good
Auricchio 2017	8	Good	Mårild 2015	9	Good	Greco 1988	6	Good
Auricchio 2020	9	Good	Marild 2017_1	9	Good	Greco 1990	7	Good
Auricchio 2021	6	Good	Marild 2018	9	Good	Güngör 2013	7	Good
Barroso 2018	9	Good	Mårild 2019_2	9	Good	Hansen 2007	8	Good
Bendersky 2020	9	Good	Meijer 2022	8	Good	Isikay 2014	9	Good
Beser 2019	8	Good	Miranda 2017	9	Good	Ivarsson 2002	9	Good
Beyerlein 2017	8	Good	Moos 2020	9	Good	Kahrs 2019	9	Good
Bingley 2004	8	Good	Moyer 2018	7	Good	Kakleas 2010	8	Good
Boechler 2023	9	Good	Naddei 2022	7	Good	KeceliBasaran 2021	8	Good
Calderoni 2016	6	Good	Namatovu 2014	9	Good	Lebwohl 2012	8	Good
Canova 2014	9	Good	Namatovu 2014_2	8	Good	Lebwohl 2014	9	Good
Canova 2015	8	Good	Namatovu 2016	7	Good	Lindfors 2019	9	Good
Capriati 2015	7	Good	Namatovu 2016_2	8	Good	Lionetti 2020	9	Good
Castellaneta 2015	7	Good	Norris 2005	8	Good	LlorentePelayo 2021	7	Good
Cerutti 2004	8	Good	Norström 2020	8	Good	Ludvigsson 2013	9	Good
Cilleruelo 2016	6	Good	Nusier 2010	7	Good	Ludvigsson 2013_1	9	Good
Crawley 2023	7	Good	Odeh 2019	7	Good	Ludvigsson 2013_2	8	Good
Crespo-Escobar 2017	6	Good	Pariente 2019	6	Good	Maleki 2023	8	Good
Dalgic 2011	6	Good	Prosperi 2021	6	Good	Mårild 2011	9	Good
Daniel 2019	7	Good	Roberts 2009	8	Good	Marild 2013	9	Good
Davidesko 2021	9	Good	Rutz 2002	7	Good	Mårild 2013_1	9	Good
DydensborgSander 2018	9	Good	Saadah 2012	7	Good	Mårild 2016	9	Good
DydensborgSander 2019	9	Good	Saari 2015	8	Good	Marild 2017_2	9	Good
Emilsson 2015_1	9	Good	Sandberg-Bennich 2002	9	Good	Myleus 2012_1	7	Good
Gatti 2019	6	Good	Schaub 2015	8	Good	Myleus 2012_2	7	Good
Gaylord 2020	8	Good	Sevelsted 2015	9	Good	Narang 2017	7	Good
Gudeta 2022	9	Good	Simre 2016	8	Good	Oikarinen 2021	8	Good
Gutvirtz 2024	9	Good	Stahl 2020_1	7	Good	Patel 2018	8	Good
Hagopian 2017	8	Good	Stahl 2020_2	8	Good	Pelayo 2020	7	Good
HårdAfSegerstad 2022	9	Good	Steiner 2019	9	Good	Peters 2001	8	Good
HardAfSegerstad 2023	9	Good	Størdal 2013	9	Good	Renata 2022	7	Good
Hummel 2007	9	Good	Størdal 2013_2	9	Good	Roman 2010	8	Good
Inns 2021	9	Good	Tanpowpong 2013	8	Good	Segerstad 2018	8	Good
Ivarsson 2003	8	Good	Tanpowpong 2023	8	Good	Selimoğlu 2013	7	Good
Ivarsson 2003_2	9	Good	Tata 2015	8	Good	Simre 2019	8	Good
Jansen 2014	9	Good	Uusitalo 2015	8	Good	Stagi 2005	7	Good
Jansen 2016	9	Good	Uusitalo 2019	8	Good	Stene 2006	8	Good
Ji 2011	8	Good	Vajravelu 2018	8	Good	Tapia 2021	8	Good
Jølving 2021	9	Good	vanderPals 2014	8	Good	Tjernberg 2014	8	Good
Kahrs 2017	9	Good	vanderVelde 2023	8	Good	Walkowiak 2010	7	Good
Kalvandi 2021	9	Good	Vriezinga 2014	9	Good	**Study ID**	**NOS C-S Score**	**Study Quality**
Kårhus 2017	9	Good	Welander 2010	8	Good	Al-Sinani 2013	6	Good
Karur 2021	8	Good	White 2023	9	Good	Batista 2012	5	Fair
Kemppainen 2017	8	Good	Wingren 2011	8	Good	Giannotti 2001	4	Poor
Kemppainen 2017_1	9	Good	Wingren 2012_0	9	Good	Ivarsson 2013	5	Fair
Koletzko 2018	9	Good	Wingren 2012_1	7	Good	Lebwohl 2013	8	Good
Kori 2022	9	Good	Wingren 2012_2	9	Good	Sadiq 2022	6	Good
Kuja-Halkola 2017	9	Good	Wingren 2012_3	9	Good	Whyte 2013	7	Good
Laitinen 2018	8	Good	Winkler 2019	8	Good	**Study ID**	**RoB 2 Score**	**Risk of Bias**
Leonard 2023	7	Good	Yang 2017	8	Good	Crespo-Escobar 2018	4 Low; 1 Some	Some
Lewy 2009	8	Good	Yoles 2018	9	Good	Hemming-Harlo 2019	5 Low	Low
Lionetti 2017	9	Good	Zacay 2024	9	Good	Hyytinen 2017	4 Low; 1 Some	Some
Liu 2014	7	Good	Zingone 2015	8	Good	Lehtinen 2016	4 Low; 1 Some	Some
						Lionetti 2014	4 Low; 1 Some	Some
						Savilahti 2018	4 Low; 1 Some	Some

### Results of meta-analyses

**Fig 2:**
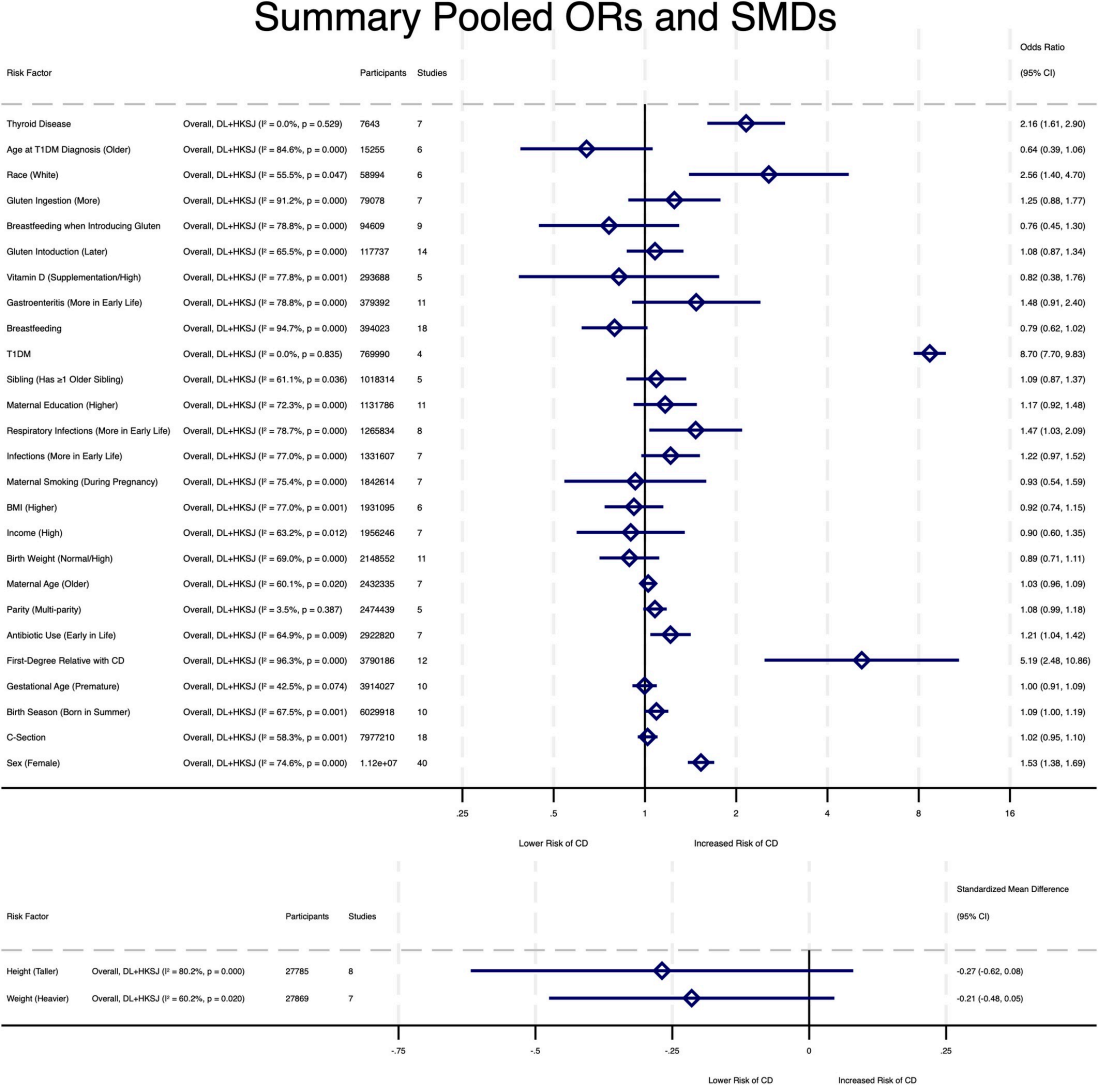
Summary forest plot for predictive factors included in meta-analysis. Upper region of plot displays pooled odds ratio estimates and lower region of plot displays standardized mean difference.

Meta-analyses of the individual risk factors are presented in Table 5 and summary forest plot Fig 2, individual forest plots are in Figures A1-A5 in S1 File. The following factors were statistically significant: having type 1 diabetes (OR 8.70, 95% CI 7.70–9.83, I^2^ = 0.0%), having a first degree relative with coeliac disease (OR 5.19, 95%CI 2.48–10.86, I^2^ = 96.3%), being of white ethnicity (OR 2.56, 95%CI 1.40–4.70, I^2^ = 55.5%), having thyroid disease (OR 2.16, 95%CI 1.61–2.90, I^2^ = 0.0%), being female (OR 1.53, 95%CI 1.39–1.69, I^2^ = 74.6%), antibiotic use in early childhood (OR 1.21, 95%CI 1.04–1.42, I^2^ = 64.9%), having frequent respiratory infections in early childhood (OR 1.47, 95%CI 1.03–2.09, I^2^ = 78.7%), and being born in the summer (OR 1.09, 95%CI 1.00–1.19, I^2^ = 67.5%).

**Table 5:** Sensitivity analysis of meta-analysis for each predictive factor comparing Hartung-Knapp-Sidik-Jonkman (HKSJ) and DerSimonian and Laird (DL) odds ratio (OR) estimates or standardized mean difference (SMD) and if they were significant for each predictive factor. Significance assessed as p≤0.05.

Predictive Factor	# of studies	# of participants	HKSJ OR (95%CI)	Significant	DL OR (95%CI)	Significant
Sex (Female)	40	11,232,409	1.53 (1.38, 1.69)	Yes	1.53 (1.40, 1.64)	Yes
Breastfeeding (any)	18	394,023	0.79 (0.62, 1.02)	No	0.79 (0.72, 0.88)	Yes
C-section	18	7,977,210	1.02 (0.95, 1.10)	No	1.02 (0.96, 1.09)	No
Gluten Introduction (Later)	14	117,737	1.08 (0.87, 1.34)	No	1.08 (0.91, 1.29)	No
First-degree relative with Celiac Disease	12	3,790,186	5.19 (2.48, 10.86)	Yes	5.19 (2.64, 10.21)	Yes
Infections (More in Early Life)	7	1,331,607	1.22 (0.97, 1.52)	No	1.22 (1.06, 1.39)	Yes
Maternal Education (Higher)	11	1,131,786	1.17 (0.92, 1.48)	No	1.19 (0.96, 1.47)	No
Birth Season (Born in Summer)	10	6,029,918	1.09 (1.00, 1.19)	Yes	1.09 (1.03, 1.15)	Yes
Respiratory Infections (More in Early Life)	8	1,265,834	1.47 (1.03, 2.09)	Yes	1.47 (1.15, 1.87)	Yes
Antibiotic Use (Early in Life)	7	2,922,820	1.21 (1.04, 1.42)	Yes	1.21 (1.10, 1.35)	Yes
Gastroenteritis (More in Early Life)	11	379,392	1.48 (0.91, 2.40)	No	1.48 (1.13, 1.92)	Yes
Breastfeeding when Introducing Gluten	9	94,609	0.76 (0.45, 1.30)	No	0.76 (0.51, 1.13)	No
Gluten Ingestion (More)	7	79,078	1.25 (0.88, 1.77)	No	1.25 (1.06, 1.48)	Yes
BMI (Higher)	6	1,931,095	0.92 (0.74, 1.15)	No	0.92 (0.84, 1.01)	No
Birth Weight (Normal/High)	11	2,148,552	0.89 (0.71, 1.11)	No	0.89 (0.78, 1.01)	No
Age at T1DM Diagnosis (Older)	6	15,255	0.64 (0.39, 1.06)	No	0.64 (0.47, 0.88)	Yes
Gestational Age (Premature)	10	3914027	1.00 (0.91, 1.09)	No	1.00 (0.91, 1.09)	No
Race (White)	6	58,994	2.56 (1.40, 4.70)	Yes	2.56 (1.65, 3.97)	Yes
Maternal Age (Older)	7	2,432,335	1.03 (0.96, 1.09)	No	1.03 (0.99, 1.06)	No
Thyroid Disease	7	7,643	2.16 (1.61, 2.90)	Yes	2.16 (1.67, 2.79)	Yes
Income (High)	7	1,956,246	0.90 (0.60, 1.35)	No	0.90 (0.69, 1.17)	No
Maternal Smoking (During Pregnancy)	7	1,842,614	0.93 (0.54, 1.59)	No	0.93 (0.64, 1.35)	No
Sibling (has ≥1 Older Sibling)	5	1,018,314	1.09 (0.87, 1.37)	No	1.09 (0.93, 1.28)	No
Vitamin D (Supplementation/High)	5	293,688	0.82 (0.38, 1.76)	No	0.82 (0.51, 1.32)	No
Parity (Multi-parity)	5	2,474,439	1.08 (0.86, 1.36)	No	1.08 (0.94, 1.24)	No
T1DM	4	769,990	8.70 (7.70, 9.83)	Yes	8.70 (7.56, 10.01)	Yes
**Predictive Factor**	**# of studies**	**# of participants**	**HKSJ SMD (95%CI)**	**Significant**	**DL SMD (95%CI)**	**Significant**
Height (Taller)	8	27,785	-0.27 (-0.62, 0.08)	No	-0.27 (-0.55, 0.01)	No
Weight (Heavier)	7	27,869	-0.21 (-0.48, 0.05)	No	-0.21 (-0.41, -0.02)	Yes

Sensitivity analysis comparing the HKSJ and DL random effects models (Table 4) found six additional predictive factors to be significant when using DL but not with HKSJ. These were: older age at T1DM diagnosis (protective, OR 0.64, 95%CI 0.47–0.88, p = 0.005, I^2^ = 84.6%), breast feeding (protective, OR 0.79, 95%CI 0.72–0.88, p<0.001, I^2^ = 94.7%), more frequent gastroenteritis (OR 1.48, 95%CI 1.13–1.92, p = 0.004, I^2^ = 78.8%), greater gluten ingestion (OR 1.25, 95%CI 1.06–1.48, p = 0.008, I^2^ = 91.2%), and more frequent infections (OR 1.22, 95%CI 1.06–1.39, p = 0.005, I^2^ = 77.0%). A heavier weight was associated with a lower risk of CD (SMD -0.21, 95%CI -0.41 to -0.02, p = 0.029, I^2^ = 60.2%) representing a small effect size [38].

The remaining factors were all found to be not significant in their respective meta-analyses including in sensitivity analysis comparing HKSJ and DL. Breast feeding at gluten introduction, vitamin D supplementation, higher birth weight, and higher maternal education all had point OR estimates suggesting a possible trend but wide confidence intervals encompassing the line of no effect (OR of 1). Higher income, higher BMI, maternal smoking, premature gestational age, being born via c-section, higher maternal age, multi-parity, age at gluten introduction and having ≥1 older sibling were all found to be non-significant and had pooled OR estimates 0.9–1.1. Taller height had a point estimate of SMD similar to weight which would show a lower risk of CD, but the confidence interval included zero.

Sensitivity analysis restricting to studies with >100 participants did not change the results (results not shown). Excluding hazard ratios and relative risks made summer season of birth not significant with a confidence interval encompassing 1, and did not meaningfully change the results for any other predictive factor (including those with pooled OR estimates >2). Restricting to only adjusted measures likewise did not change the results for those factors with ≥3 studies remaining to proceed with meta-analysis. As most studies were of good or fair quality, restricting analysis to only studies of good quality did not change the results for those factors with ≥3 studies remaining to proceed with meta-analysis. No studies considered to be of poor quality/high risk of bias were included in the main meta-analyses.

### Reporting biases

Funnel plots are shown in Figures A6-A10 in S1 File. For height and weight, Egger’s test was not significant. Most predictive factors had reasonably symmetric funnel plots except for first degree relative with CD, infections, gluten ingestion, age at T1DM diagnosis, respiratory infections, and antibiotics suggestive of potential publication bias or selective outcome reporting for these factors.

## Discussion

### Principal findings

To our knowledge, this is the only systematic review of studies examining the association between predictive factors relevant to clinicians seeing initial presentation of children and the risk of CD specifically in children and young people. This systematic review compiled evidence from the peer-reviewed literature to identify 145 factors assessed for an association with a diagnosis of CD. Meta-analyses of the 28 most reported predictive factors revealed 14 factors to be significant on one or both of the random effects models and quantified the strength of these associations. The remaining 14 factors were not significantly associated with CD using either model. Of the factors found to be significant, five had OR estimates >1.5, and the remaining all had smaller effect sizes.

### Comparison with previous literature

Individual predictive factors have been summarized in other reviews, such as the significance of breast feeding [39–46], infant feeding [47], perinatal factors [48] or gluten introduction [49]. Some reviews have approached the question of predictive factors for CD more broadly [15, 50, 51]. A comprehensive systematic review and meta-analysis was recently published by Elwenspoek et al. on the “diagnostic indicators for coeliac disease” [15]. This review included both adults and children and included symptoms [15]. Our review differs in that it excluded symptoms and adds to the work of Elwenspoek et al. by including a broader array of potential predictive factors, such as perinatal risk factors and age at gluten introduction). Both their review and this one restricted meta-analysis to factors with ≥5 studies. The results are consistent between our meta-analysis previous reviews in the significant association of T1DM, thyroid disease and family history of CD [15, 52–54].

Family history of CD and autoimmune conditions are well-documented risk factor given the genetic/heritable factors associated with CD [55], consistent with our findings for family history, T1DM and thyroid disease as predictive factors for CD [55]. These are all early indicators which could be used as criteria for screening. Multiple autoimmune conditions are more common in female patients, which is consistent with our finding of being female as a predictive factor [56]. The effect of ethnicity may be explained through the prevalence of genetic factors in certain populations, and by other non-genetic factors such as by socioeconomic status or day care attendance as shown in other studies, so it needs to be considered in context [57].

This analysis has found that early life antibiotic exposure and respiratory infections are both associated with CD, and having more infections was found to be significantly associated using the DL model. Some reports have suggested mechanisms for how antibiotics and infections could increase the risk of CD [58, 59]. The reports varied in how antibiotic use and respiratory infections were defined but they all focused on early childhood. The proposed mechanisms relate to the impact of infections and antibiotics on the composition of the gut microbiome, the regulation of the immune system by certain infections and how some bacterial peptides may mimic immunogenic gluten peptides, priming the immune system to respond to gluten [58, 59]. This impact of infections may also explain why season/month of birth is associated with CD in this analysis, as children born in the summer experience their first viral season/infections at a different age than children born in the winter [60].

Six additional factors (older age at T1DM diagnosis, being breast fed, more frequent infections, increased amount of gluten ingestion, more frequent gastroenteritis, and heavier weight) had significant associations in sensitivity analyses using the DL random effects models. Some of the primary studies included in the meta-analyses reported significant associations [20, 21, 61–82]. Given their lack of significance in the primary analysis using the HKSJ random effects model, there remains uncertainty about the strength of these associations, and these factors may be candidates for further research to address this uncertainty. Lower height and/or weight may be manifestations of CD rather than early indicators and may suggest late diagnosis or failure to thrive. However some evidence suggests growth rate may be affected before any other symptoms or disease manifestations are present so in some children may be a useful indicator [83].

There is uncertainty in the evidence as to whether breastfeeding is protective against CD or if it delays onset or changes the symptom profile at presentation. Breastfeeding has been the subject of previous reviews [43, 84]. One paper included in the meta-analysis for breastfeeding found a higher rate of CD amongst children breastfed for ≥4 months [20]. This study only included children with T1DM, and we had to calculate an unadjusted odds ratio from the raw prevalence data included in the paper, so it is likely that this association may be explained by other factors as all other studies found breastfeeding to be protective or to have no significant effect.

### Strengths & limitations

Our review extends what is known by compiling evidence in a systematic review and meta-analysiss, identifying predictive factors and strengths of associations. Our work aligns with the James Lind Alliance and Coeliac UK research priorities by summarising the current knowledge on the risk factors for the development of CD, and by helping to better understand the association between CD and other conditions [23].

We utilised a comprehensive search strategy including forward and backward citation searching of included studies and relevant reviews to capture all published articles relating to the study question. Screening and data extraction was performed in duplicate and strict inclusion criteria were applied to minimize bias.

The clinical and methodological diversity between studies can be observed in the reported I^2^ statistics which are often indicative of moderate to high heterogeneity [85]. The direction of associations was typically aligned across studies for the same predictive factor despite the heterogeneity. We anticipated there may be heterogeneity and, therefore, chose to use a random effects model [85]. We chose the HKSJ random effects model as our primary form of meta-analysis which performs better in simulation models than DL and is less likely to overestimate possible effects given the wider confidence intervals [31].

As demonstrated by the instances of funnel plot asymmetry, there is a possibility of some publication and/or selective outcome reporting bias. We were limited in our ability to perform subgroup analyses (such as by sex) because insufficient studies reported stratified data. Sensitivity analyses restricting to larger study size (>100 participants) and fair to high quality studies were performed when possible and they did not change the results from the primary analyses which offers confidence in the results of these meta-analyses.

Differences in definitions and cut-offs of some predictive factors used in the primary studies could have contributed to the heterogeneity in study results in the meta-analysis and may have limited the ability to fully evaluate the impact of some factors. For example, breast feeding was assessed across studies from between 30 days of breast feeding to >12 months. To combine these studies together, we simplified the definition to a dichotomous variable of ‘any breast feeding’ compared to ‘no breast feeding’. Some studies included routinely collected data which presents the limitation of the likely under-ascertainment of diagnosis. The cohorts and cases/controls used in many studies recruited people with known diagnoses or specific risk factors or from specific clinical settings which can introduce a bias about the applicability of those results to the larger population.

We combined OR, HR, and RR together in the meta-analyses given the range of effect measures reported in the primary studies. The difference between these effect measures is minimal when the outcome is rare, as was the case with the factors included in the meta-analyses, but is more pronounced when the association is greater/when the event is common [32, 33]. Including hazard ratios and relative risks in calculating a combined odds ratio potentially underestimates the overall effect as odds ratios will exceed both relative risks and hazard ratios when greater than one [33]. We also combined adjusted and unadjusted effect measures (preferring adjusted measures when they were available) [32]. Sensitivity analysis restricting to only OR and to only adjusted measures were performed when possible and did not change the overall conclusions. Although there is a risk of bias, the results of our sensitivity analysis provide confidence in the reported findings.

### Clinical implications

Predictive factors can be included in clinical guidelines to guide testing and clinical decision making. Clinical guidelines on CD typically focus on signs and symptoms, sometimes alongside predictive factors/associated conditions, such as T1DM, thyroid disease and having a first degree relative with CD [8–11]. These factors were all found to have significant and strong associations in this review, adding to the evidence supporting these guidelines.

Our meta-analyses have not identified other factors which could be easily used to risk stratify in isolation but have identified factors which may be suitable candidates for future research. The other factors identified in this review as significant (being female, being of white ethnicity, having been breast fed, history of early childhood antibiotic use and frequent/severe infections etc.), are easy to assess in an initial clinical assessment and may have greater value as predictive factors in the context of specific symptoms or when combined. Future studies could investigate the feasibility and predictive value of combining candidate factors which can be assessed in primary care consultation with signs and symptoms to aid in risk stratification. These factors and symptoms together could be used to create clinical risk prediction tools or automated clinical prompts for use in primary care. Children with CD can experience a range of non-classical CD symptoms [86]; these children are more likely to experience delayed diagnosis, so factors which aid in identifying at-risk children prior to diagnosis would be of particular benefit to this population [87–89].

Diagnostic delay for CD in children may range from months to years from symptom onset with an average delay of 5 months; there is evidence that when diagnosis is delayed longer than three years, children have lower body weight and shorter stature at the time of diagnosis [89, 90]. Previous misdiagnosis and certain symptom patterns associated with longer delays and female children and children at older ages were also more likely to have a delayed diagnosis [90]. These findings highlight an opportunity for further research to identify children with patterns of symptoms and predictive factors at risk for longer delays in diagnosis for targeted testing.

Ultimately, the value of understanding and utilising predictive factors for CD lies in the utility of knowing which factors are and which are not associated with CD. This can guide clinicians as to what specific information is useful to assess in a time-limited setting and to help them to identify children at increased risk of CD and the need for testing.

Some of the significant predictive factors included in our meta-analyses are modifiable risk factors (breast feeding, antibiotic use, and gluten ingestion). Although these cannot be modified for an individual patient at the time of screening, these can be considered along with other evidence to support recommendations and guidance regarding advice to parents on breast feeding and diet, and antibiotic prescribing in early childhood.

## Conclusion

Consistent with previous systematic reviews, this systematic review and meta-analysis found that children with T1DM, thyroid disease or a family history of CD are at an increased risk of CD and quantified the strength of those associations with the most recently available evidence across studies. This would suggest these children should be considered at high risk and offered testing. In addition to reinforcing these previously identified factors, this systematic review and meta-analysis revealed additional predictive factors and factors of significance worthy of assessment and consideration in the determination of testing for CD. Female sex, white ethnicity, age at T1DM diagnosis, breast feeding, more frequent infections (including specifically respiratory infections and gastroenteritis), lower weight at assessment (including poor weight gain or dropping centiles), antibiotic use, amount of gluten ingestion and being born in the summer may have some associations with CD. These are candidate factors for future research combining predictive factors and/or symptoms for risk stratification. The modifiable factors for CD in children include breast feeding, antibiotic use and gluten ingestion, which may be relevant for population health recommendations and guidelines on these factors.

## Supporting information

S1 File Tables A1–A5, Document A1, and Figures A1–A10. (DOCX)

S2 File Includes all risk of bias assessments. (XLSX)

S1 Checklist Completed PRISMA checklist for submitted manuscript. (DOCX)
