# Supplementary material for: Predictive factors for the diagnosis of coeliac disease in children and young people in primary care: A systematic review and meta-analysis
Source: PLoS One. 2024 Dec 20;19(12):e0306844. doi: 10.1371/journal.pone.0306844 (PMC11661599; doi:10.1371/journal.pone.0306844)
Supplement: S1 File — (DOCX) [file pone.0306844.s001.docx]

# Supplementary Figures/Tables

Table A1

Search strategy designed and used in Ovid MEDLINE®. Original search run 25^th^ May 2022 was for CD, T1DM and IBD. When search was updated April 2023, Lines 1-7 and 10-15 were omitted to focus solely on CD. Search strategy was adapted for the other databases included in the search: Embase (OvidSP)[1974-present], Cochrane Database of Systematic Reviews, and Cochrane Central Register of Controlled Trials.

|  | [Medline (Ovid MEDLINE® Epub Ahead of Print, In-Process & Other Non-Indexed Citations, Ovid MEDLINE® Daily and Ovid MEDLINE®) 1946 to present](https://ovidsp.ovid.com/ovidweb.cgi?T=JS&NEWS=N&PAGE=main&SHAREDSEARCHID=3hRkZZYN6rQm1YL3YW84VS8enhsaOJlDWxR11JV4mzszVntZVS7VlBeb2FM2AQJB0) |  |
| --- | --- | --- |
| 1 | Diabetes Mellitus, Type 1/ | 82654 |
| 2 | ((type 1 or type i) adj diabetes).ti,ab,kf. | 51776 |
| 3 | diabetic ketoacidosis.ti,ab,kf. | 6762 |
| 4 | (T1DM or TIDM).ti,ab,kf. | 5792 |
| 5 | insulin dependent diabetes.ti,ab,kf. | 20823 |
| 6 | IDDM.ti,ab,kf. | 6892 |
| 7 | childhood diabetes.ti,ab,kf. | 868 |
| 8 | Celiac Disease/ | 21355 |
| 9 | c?eliac disease.ti,ab,kf. | 19744 |
| 10 | exp Inflammatory Bowel Diseases/ | 91561 |
| 11 | inflammatory bowel disease.ti,ab,kf. | 51787 |
| 12 | IBD.ti,ab,kf. | 31342 |
| 13 | ulcerative colitis.ti,ab,kf. | 45330 |
| 14 | UC.ti,ab,kf. | 24614 |
| 15 | Crohn*.ti,ab,kf. | 53470 |
| 16 | or/1-15 | 271923 |
| 17 | *protective factors/ or *risk factors/ | 1508 |
| 18 | (risk adj4 factor?).ti,ab,kf. | 725984 |
| 19 | (protective adj4 factor?).ti,ab,kf. | 28013 |
| 20 | or/17-19 | 740348 |
| 21 | adolescent/ or exp child/ or young adult/ | 3714576 |
| 22 | (child* or schoolchild* or "school age*" or pediatric* or paediatric* or toddler* or preschool* or pre-school* or infant* or baby or babies).ti,ab,kf. | 2112875 |
| 23 | young men.ti,ab,kf. | 15643 |
| 24 | young women.ti,ab,kf. | 25415 |
| 25 | young male?.ti,ab,kf. | 15511 |
| 26 | young female?.ti,ab,kf. | 10494 |
| 27 | young adult?.ti,ab,kf. | 106409 |
| 28 | young people.ti,ab,kf. | 32907 |
| 29 | adolescen*.ti,ab,kf. | 343163 |
| 30 | teen*.ti,ab,kf. | 33796 |
| 31 | youth.ti,ab,kf. | 85638 |
| 32 | or/21-31 | 4665220 |
| 33 | 16 and 20 and 32 | 4510 |
|  |  |  |
|  | Update search - Apr 2023 (& same terms used Apr 2024) |  |
|  | [Medline (Ovid MEDLINE® Epub Ahead of Print, In-Process & Other Non-Indexed Citations, Ovid MEDLINE® Daily and Ovid MEDLINE®) 1946 to present](https://ovidsp.ovid.com/ovidweb.cgi?T=JS&NEWS=N&PAGE=main&SHAREDSEARCHID=5jEUrJUVqzrbrrehW66AJFr0iKrXFHm6ms7TuT4tXsIxIKKUn79GA8fjGwEOsFgiI) |  |
| 1 | Celiac Disease/ | 21771 |
| 2 | c?eliac disease.ti,ab,kf. | 20465 |
| 3 | or/1-2 | 26764 |
| 4 | *protective factors/ or *risk factors/ | 1538 |
| 5 | (risk adj4 factor?).ti,ab,kf. | 781269 |
| 6 | (protective adj4 factor?).ti,ab,kf. | 31506 |
| 7 | or/4-6 | 797338 |
| 8 | adolescent/ or exp child/ or young adult/ | 3797125 |
| 9 | (child* or schoolchild* or "school age*" or pediatric* or paediatric* or toddler* or preschool* or pre-school* or infant* or baby or babies).ti,ab,kf. | 2212239 |
| 10 | young men.ti,ab,kf. | 16241 |
| 11 | young women.ti,ab,kf. | 26605 |
| 12 | young male?.ti,ab,kf. | 16317 |
| 13 | young female?.ti,ab,kf. | 11154 |
| 14 | young adult?.ti,ab,kf. | 113976 |
| 15 | young people.ti,ab,kf. | 35807 |
| 16 | adolescen*.ti,ab,kf. | 365903 |
| 17 | teen*.ti,ab,kf. | 35360 |
| 18 | youth.ti,ab,kf. | 92640 |
| 19 | or/8-18 | 4799176 |
| 20 | 3 and 7 and 19 | 353 |

|  | [Embase 1974 to present](https://ovidsp.ovid.com/ovidweb.cgi?T=JS&NEWS=N&PAGE=main&SHAREDSEARCHID=22yfa8aKuuz27vzDoaoKS1lAoHAMkZMzLUUFEWjE3Ixs8IWqchuw7DrOVEzTGelfD) |  |
| --- | --- | --- |
| 1 | *insulin dependent diabetes mellitus/ | 78553 |
| 2 | ((type 1 or type i) adj diabetes).ti,ab,kf. | 81717 |
| 3 | diabetic ketoacidosis.ti,ab,kf. | 10876 |
| 4 | (T1DM or TIDM).ti,ab,kf. | 11224 |
| 5 | insulin dependent diabetes.ti,ab,kf. | 25252 |
| 6 | IDDM.ti,ab,kf. | 8172 |
| 7 | childhood diabetes.ti,ab,kf. | 1154 |
| 8 | *celiac disease/ | 21107 |
| 9 | c?eliac disease.ti,ab,kf. | 28811 |
| 10 | exp *inflammatory bowel disease/ | 106573 |
| 11 | inflammatory bowel disease.ti,ab,kf. | 87946 |
| 12 | IBD.ti,ab,kf. | 64870 |
| 13 | ulcerative colitis.ti,ab,kf. | 73166 |
| 14 | UC.ti,ab,kf. | 50660 |
| 15 | Crohn*.ti,ab,kf. | 92778 |
| 16 | or/1-15 | 365539 |
| 17 | *risk factor/ | 103577 |
| 18 | (risk adj4 factor?).ti,ab,kf. | 1075547 |
| 19 | (protective adj4 factor?).ti,ab,kf. | 36237 |
| 20 | or/17-19 | 1110445 |
| 21 | exp adolescent/ or exp child/ or young adult/ | 3977186 |
| 22 | (child* or schoolchild* or "school age*" or pediatric* or paediatric* or toddler* or preschool* or pre-school* or infant* or baby or babies).ti,ab,kf. | 2602513 |
| 23 | young men.ti,ab,kf. | 19007 |
| 24 | young women.ti,ab,kf. | 34617 |
| 25 | young male?.ti,ab,kf. | 19802 |
| 26 | young female?.ti,ab,kf. | 14270 |
| 27 | young adult?.ti,ab,kf. | 140459 |
| 28 | young people.ti,ab,kf. | 44876 |
| 29 | adolescen*.ti,ab,kf. | 430090 |
| 30 | teen*.ti,ab,kf. | 46380 |
| 31 | youth.ti,ab,kf. | 99906 |
| 32 | or/21-31 | 4836622 |
| 33 | 16 and 20 and 32 | 5953 |

|  | Update search - Apr 2023 (& same terms used Apr 2024) |  |
| --- | --- | --- |
|  |  |  |
|  | [Embase 1974 to present](https://ovidsp.ovid.com/ovidweb.cgi?T=JS&NEWS=N&PAGE=main&SHAREDSEARCHID=1Y0I2MRLAWI5esrJSlP0Pe5Tn8ezhOjFqizxrDAvmSch5uiZnAVdArnIWNtvjaRqu) |  |
| 1 | *celiac disease/ | 22205 |
| 2 | c?eliac disease.ti,ab,kf. | 30618 |
| 3 | or/1-2 | 32713 |
| 4 | *risk factor/ | 123193 |
| 5 | (risk adj4 factor?).ti,ab,kf. | 1180741 |
| 6 | (protective adj4 factor?).ti,ab,kf. | 41441 |
| 7 | or/4-6 | 1220708 |
| 8 | exp adolescent/ or exp child/ or young adult/ | 4224413 |
| 9 | (child* or schoolchild* or "school age*" or pediatric* or paediatric* or toddler* or preschool* or pre-school* or infant* or baby or babies).ti,ab,kf. | 2791705 |
| 10 | young men.ti,ab,kf. | 20004 |
| 11 | young women.ti,ab,kf. | 36776 |
| 12 | young male?.ti,ab,kf. | 21055 |
| 13 | young female?.ti,ab,kf. | 15356 |
| 14 | young adult?.ti,ab,kf. | 153891 |
| 15 | young people.ti,ab,kf. | 49672 |
| 16 | adolescen*.ti,ab,kf. | 468987 |
| 17 | teen*.ti,ab,kf. | 49368 |
| 18 | youth.ti,ab,kf. | 110351 |
| 19 | or/8-18 | 5133786 |
| 20 | 3 and 7 and 19 | 559 |

| ID | [Cochrane](https://ovidsp.ovid.com/ovidweb.cgi?T=JS&NEWS=N&PAGE=main&SHAREDSEARCHID=22yfa8aKuuz27vzDoaoKS1lAoHAMkZMzLUUFEWjE3Ixs8IWqchuw7DrOVEzTGelfD) Library Search |  |
| --- | --- | --- |
| #1 | MeSH descriptor: [Diabetes Mellitus, Type 1] this term only |  |
| #2 | ((("type 1" or "type i" or "insulin dependent" or childhood) adj diabetes) or t1dm or tidm or iddm or "diabetic ketoacidosis"):ti,ab,kw |  |
| #3 | MeSH descriptor: [Celiac Disease] explode all trees |  |
| #4 | (coeliac or celiac):ti,ab,kw |  |
| #5 | MeSH descriptor: [Inflammatory Bowel Diseases] explode all trees |  |
| #6 | ("inflammatory bowel disease" or ibd or "ulcerative colitis" or uc or crohn*):ti,ab,kw |  |
| #7 | #1 or #2 or #3 or #4 or #5 or #6 |  |
| #8 | MeSH descriptor: [Risk Factors] explode all trees |  |
| #9 | MeSH descriptor: [Protective Factors] explode all trees |  |
| #10 | (((risk or protective) NEAR/4 factor*)):ti,ab,kw |  |
| #11 | #8 or #9 or #10 |  |
| #12 | MeSH descriptor: [Child] explode all trees |  |
| #13 | MeSH descriptor: [Adolescent] explode all trees |  |
| #14 | MeSH descriptor: [Young Adult] explode all trees |  |
| #15 | (child* or schoolchild* or "school age*" or pediatric* or paediatric* or toddler* or preschool* or pre-school* or infant* or baby or babies or teen* or adolescen* or youth or (young NEXT (men or women or male* or female or adult* or people))):ti,ab,kw |  |
| #16 | #12 or #13 or #14 or #15 |  |
| #17 | #7 and #11 and #16 |  |
|  | Update search - Apr 2023 (& same terms used Apr 2024)  [Cochrane](https://ovidsp.ovid.com/ovidweb.cgi?T=JS&NEWS=N&PAGE=main&SHAREDSEARCHID=22yfa8aKuuz27vzDoaoKS1lAoHAMkZMzLUUFEWjE3Ixs8IWqchuw7DrOVEzTGelfD) Library |  |
| ID | Search | Hits |
| #1 | MeSH descriptor: [Celiac Disease] explode all trees | 500 |
| #2 | (coeliac or celiac):ti,ab,kw | 1470 |
| #3 | #1 or #2 | 1470 |
| #4 | MeSH descriptor: [Risk Factors] explode all trees | 33011 |
| #5 | MeSH descriptor: [Protective Factors] explode all trees | 222 |
| #6 | (((risk or protective) NEAR/4 factor*)):ti,ab,kw | 83634 |
| #7 | #4 or #5 or #6 | 83634 |
| #8 | MeSH descriptor: [Child] explode all trees | 77476 |
| #9 | MeSH descriptor: [Adolescent] explode all trees | 125165 |
| #10 | MeSH descriptor: [Young Adult] explode all trees | 84504 |
| #11 | (child* or schoolchild* or "school age*" or pediatric* or paediatric* or toddler* or preschool* or pre-school* or infant* or baby or babies or teen* or adolescen* or youth or (young NEXT (men or women or male* or female or adult* or people))):ti,ab,kw | 393952 |
| #12 | #8 or #9 or #10 or #11 | 393952 |
| #13 | #3 and #7 and #12 | 27 |

Document A1

## **Adapted Newcastle-Ottawa Scale for Cross-Sectional Studies**

**Selection: (Maximum of 4 stars)**

1. **Representativeness of the sample:**
2. Truly representative of the average in the target population. **(all subjects or random sampling)** *
3. Somewhat representative of the average in the target population. **(non-random sampling**) *
4. Selected group of users.
5. No description of the sampling strategy.
6. **Sample size:**
7. Justified and satisfactory.*
8. Not justified.
9. **Ascertainment of exposure:**
10. Validated measurement tool.*
11. Non-validated measurement tool, but the tool is available or described.*
12. No description of the measurement tool.
13. **Non-respondents:**
14. Comparability between respondents and non-respondents characteristics is established, and the response rate is satisfactory.*
15. The response rate is unsatisfactory, or the comparability between respondents and non-respondents is unsatisfactory.
16. No description of the response rate or the characteristics of the responders and the non-responders.

**Comparability**: **(Maximum of 2 stars)**

1. **The subjects in different outcome groups are comparable, based on the study design or analysis. Confounding factors are controlled:**
2. The study controls for the most important factor (age).*
3. The study controls for at least one additional factor on the following list.*

**Outcome: (Maximum of 2 stars)**

1. **Assessment of outcome:**
2. Independent blind assessment.*
3. Record linkage.*
4. Self-report.*
5. No description.
6. **Statistical test:**
7. The statistical test used to analyze the data is clearly described and appropriate, and the measurement of the association is presented, including confidence intervals or probability level (p-value).*
8. The statistical test is not appropriate, not described, or incomplete.

Adapted from:

Moskalewicz A, Oremus M. No clear choice between Newcastle–Ottawa Scale and Appraisal Tool for Cross-Sectional Studies to assess methodological quality in cross-sectional studies of health-related quality of life and breast cancer. Journal of clinical epidemiology. 2020 Apr 1;120:94-103.

Table A2: References for Included Reports

| Extracted Study ID (Author Year) | Reference |
| --- | --- |
| Ahmed 2022 | Ahmed F, Al Jneibi S, Rajah J, Al Remeithi S. Time trend and potential risk factors for celiac disease development in children with type 1 diabetes mellitus: 10-year single center experience in the Emirate of Abu Dhabi-UAE. Pediatr Diabetes. 2022;23(Supplement 31):123. |
| Al-Hussaini 2012 | Al-Hussaini A, Sulaiman N, Al-Zahrani M, Alenizi A, El Haj I. High prevalence of celiac disease among Saudi children with type 1 diabetes: a prospective cross-sectional study. BMC Gastroenterol. 2012;12(1):1-7. |
| Al-Sinani 2013 | Al-Sinani S, Sharef SW, Al-Yaarubi S, Al-Zakwani I, Al-Naamani K, Al-Hajri A, et al. Prevalence of Celiac Disease in Omani Children with Type 1 Diabetes Mellitus: A Cross Sectional Study. Oman medical journal. 2013;28(4):260-3. |
| Ascher 1997 | Ascher H, Krantz I, Rydberg L, Nordin P, Kristiansson B. Influence of infant feeding and gluten intake on coeliac disease. Arch Dis Child. 1997;76(2):113-7. |
| Assa 2018 | Assa A, Waisbourd-Zinman O, Daher S, Shamir R. Birth Month as a Risk Factor for the Diagnosis of Celiac Disease Later in Life: A Population-based Study. Journal of pediatric gastroenterology and nutrition. 2018;67(3):367-70. |
| Auricchio 1983 | Auricchio S, D F, G dR, Giunta A, Marzorati D, Prampolini L, et al. Does Breast Feeding Protect Against the Development of Clinical Symptoms of Celiac Disease in Children. Journal of Pediatric Gastroenterology and Nutrition. 1983;2(3):428-33. |
| Auricchio 2021 | Auricchio R, Calabrese I, Galatola M, Cielo D, Carbone F, Mancuso M, et al. Gluten ingestion in the first years of life is a risk factor for celiac disease in children genetically predisposed. Journal of pediatric gastroenterology and nutrition. 2021;72(SUPPL 1):24. |
| Batista 2012 | Batista IC, Gandolfi L, Nobrega YKM, Almeida RC, Almeida LM, Campos Junior D, et al. Autism spectrum disorder and celiac disease: no evidence for a link. Arquivos de Neuro-psiquiatria. 2012;70:28-33. |
| Beser 2019 | Beser OF, Gulluelli E, Cullu Cokugras F, Erkan T, Kutlu T, Yagci RV, et al. Prevalence and Clinical Features of Celiac Disease in Healthy School-Aged Children. Dig Dis Sci. 2019;64(1):173-81. |
| Beyerlein 2017 | Beyerlein A, Donnachie E, Ziegler A-G. Infections in Early Life and Development of Celiac Disease. American journal of epidemiology. 2017;186(11):1277-80. |
| Bingley 2004 | Bingley PJ, Norcross AJ, Lock RJ, Ness AR, Jones RW. Undiagnosed coeliac disease at age seven: population based prospective birth cohort study. Bmj. 2004;328(7435):322-3. |
| Bittker 2019 | Bittker SS, Bell KR. Potential risk factors for celiac disease in childhood: a case-control epidemiological survey. Clinical and experimental gastroenterology. 2019;12:303-19. |
| Boechler 2023 | Boechler M, Susi A, Hisle-Gorman E, Rogers PL, Nylund CM. Acid Suppression and Antibiotics Administered during Infancy Are Associated with Celiac Disease. J Pediatr. 2023;254:61-7.e1. |
| Calderoni 2016 | Calderoni S, Santocchi E, Del Bianco T, Brunori E, Caponi L, Paolicchi A, et al. Serological screening for Celiac Disease in 382 pre-schoolers with Autism Spectrum Disorder. Ital. 2016;42(1):98. |
| Capriati 2015 | Capriati T, Francavilla R, Castellaneta S, Ferretti F, Diamanti A. Impact of the birth’s season on the development of celiac disease in Italy. Eur J Pediatr. 2015;174(12):1657-63. |
| Carlsson 2002 | Carlsson AK, Lindberg BA, Bredberg ACA, Hyoty H, Ivarsson S-A. Enterovirus infection during pregnancy is not a risk factor for celiac disease in the offspring. Journal of pediatric gastroenterology and nutrition. 2002;35(5):649-52. |
| Castellaneta 2015 | Castellaneta S, Piccinno E, Oliva M, Cristofori F, Vendemiale M, Ortolani F, et al. High Rate of Spontaneous Normalization of Celiac Serology in a Cohort of 446 Children With Type 1 Diabetes: A Prospective Study. Diabetes Care. 2015;38(5):760-6. |
| Cerutti 2004 | Cerutti F, Bruno G, Chiarelli F, Lorini R, Meschi F, Sacchetti C. Younger age at onset and sex predict celiac disease in children and adolescents with type 1 diabetes: an Italian multicenter study. Diabetes Care. 2004;27(6):1294-8. |
| Cilleruelo 2016 | Cilleruelo ML, Fernandez-Fernandez S, Jimenez-Jimenez J, Rayo AI, de Larramendi CH. Prevalence and Natural History of Celiac Disease in a Cohort of At-risk Children. Journal of pediatric gastroenterology and nutrition. 2016;62(5):739-45. |
| Dalgic 2011 | Dalgic B, Sari S, Ozcan B, Basturk B, Ensari A, Egritas O, et al. The evaluation of factors and symptoms related to celiac disease in Turkish children. Turk Pediatri Arsivi. 2011;46(4):314-21. |
| DydensborgSander 2018 | Dydensborg Sander S, Hansen AV, Størdal K, Andersen A-MN, Murray JA, Husby S. Mode of delivery is not associated with celiac disease. Clinical Epidemiology. 2018:323-32. |
| DydensborgSander 2019 | Dydensborg Sander S, Nybo Andersen A-M, Murray JA, Karlstad O, Husby S, Stordal K. Association Between Antibiotics in the First Year of Life and Celiac Disease. Gastroenterology. 2019;156(8):2217-29. |
| Fälth-Magnusson 1996 | Fälth-Magnusson K, Franzen L, Jansson G, Laurin P, Stenhammar L. Infant feeding history shows distinct differences between Swedish celiac and reference children. Pediatric allergy and immunology : official publication of the European Society of Pediatric Allergy and Immunology. 1996;7(1):1-5. |
| Francavilla 2011 | Francavilla R, Castellaneta S, Lionetti E, Tomarchio S, Di Mauro F, Borrelli G, et al. Mode of delivery is not associated with celiac disease: Analysis of possible risk and protective factor in a population of 14500 children. Digestive and Liver Disease. 2011;43(SUPPL. 5):S440. |
| Gatti 2019 | Gatti S, Lionetti E, Balanzoni L, Verma AK, Galeazzi T, Gesuita R, et al. Increased Prevalence of Celiac Disease in School-age Children in Italy. Clinical gastroenterology and hepatology : the official clinical practice journal of the American Gastroenterological Association. 2019;18(3):596-603. |
| Gaylord 2020 | Gaylord A, Trasande L, Kannan K, Thomas KM, Lee S, Liu M, et al. Persistent organic pollutant exposure and celiac disease: A pilot study. Environmental research. 2020;186:109439. |
| Giannotti 2001 | Giannotti A, Tiberio G, Castro M, Virgilii F, Colistro F, Ferretti F, et al. Coeliac disease in Williams syndrome. Journal of medical genetics. 2001;38(11):767-8. |
| Greco 1988 | Greco L, Auricchio S, Mayer M, Grimaldi M. Case control study on nutritional risk factors in celiac disease. Journal of pediatric gastroenterology and nutrition. 1988;7(3):395-9. |
| Greco 1990 | Greco L, De Seta L, D'Adamo G, Baldassarre C, Mayer M, Siani P, et al. Atopy and coeliac disease: bias or true relation? Acta paediatrica Scandinavica. 1990;79(6-7):670-4. |
| Gudeta 2022 | Gudeta AN, Andrén Aronsson C, Binagdie BB, Girma A, Agardh D. Incidence of celiac disease autoimmunity and associations with maternal tuberculosis and pediatric Helicobacter pylori infections in 4-year-old Ethiopian children followed up in an HLA genotyped birth cohort. Frontiers in Pediatrics. 2022;10:999287. |
| Güngör 2013 | Güngör S, Celiloğlu ÖS, Özcan Ö, Raif SG, Selimoğlu MA. Frequency of celiac disease in attention-deficit/hyperactivity disorder. Journal of Pediatric Gastroenterology and Nutrition. 2013;56(2):211-4. |
| Hansen 2007 | Hansen DG, Bennedbaek FN, Hansen LK, Høier-Madsen M, Hegedüs L, Jacobsen BB, et al. High prevalence of coeliac disease in Danish children with type I diabetes mellitus. Acta paediatrica (Oslo, Norway : 1992). 2007;90(11):1238-43. |
| Hemming-Harlo 2019 | Hemming-Harlo M, Lähdeaho M-L, Mäki M, Vesikari T. Rotavirus Vaccination Does Not Increase Type 1 Diabetes and May Decrease Celiac Disease in Children and Adolescents. The Pediatric infectious disease journal. 2019;38(5):539-41. |
| Hyytinen 2017 | Hyytinen M, Savilahti E, Virtanen SM, Harkonen T, Ilonen J, Luopajarvi K, et al. Avoidance of Cow's Milk-Based Formula for At-Risk Infants Does Not Reduce Development of Celiac Disease: A Randomized Controlled Trial. Gastroenterology. 2017;153(4):961-70.e3. |
| Inns 2021 | Inns T, Fleming KM, Iturriza-Gomara M, Hungerford D. Paediatric rotavirus vaccination, coeliac disease and type 1 diabetes in children: a population-based cohort study. BMC Med. 2021;19(1):147. |
| Isikay 2014 | Isikay S, Hizli S. Frequency of coeliac disease in children with breath-holding spells. Journal of paediatrics and child health. 2014;50(11):916-9. |
| Ivarsson 2013 | Ivarsson A, Myléus A, Norström F, van der Pals M, Rosén A, Högberg L, et al. Prevalence of Childhood Celiac Disease and Changes in Infant Feeding. Pediatrics. 2013;131(3):687-94. |
| Ji 2011 | Ji J, Ludvigsson JF, Sundquist K, Sundquist J, Hemminki K. Incidence of celiac disease among second-generation immigrants and adoptees from abroad in Sweden: Evidence for ethnic differences in susceptibility. Scand J Gastroenterol. 2011;46(7-8):844-8. |
| Kahrs 2019 | Kahrs CR, Chuda K, Tapia G, Stene LC, Mårild K, Rasmussen T, et al. Enterovirus as trigger of coeliac disease: nested case-control study within prospective birth cohort. BMJ (Clinical research ed). 2019;364(7):373-. |
| Kakleas 2010 | Kakleas K, Karayianni C, Critselis E, Papathanasiou A, Petrou V, Fotinou A, et al. The prevalence and risk factors for coeliac disease among children and adolescents with type 1 diabetes mellitus. Diabetes research and clinical practice. 2010;90(2):202-8. |
| Kårhus 2017 | Kårhus LL, Gunnes N, Størdal K, Bakken IJ, Tapia G, Stene LC, et al. Influenza and risk of later celiac disease: a cohort study of 2.6 million people. Scand J Gastroenterol. 2017;53(1):15-23. |
| Kalvandi 2021 | Kalvandi G, Shahramian I, Farmany A, Yadegari S, Parooie F. Serological study of celiac disease in children with dental caries. Human antibodies. 2021;29(4):237-41. |
| KeceliBasaran 2021 | Keceli Basaran M, Dogan C, Bal M, Geylani Gulec S, Urganci N. Does Having Rotavirus Infection in Early Childhood Increase the Risk of Celiac Disease? Journal of Pediatric Infectious Diseases. 2021;16(4):154-9. |
| Kori 2022 | Kori M, Topf-Olivestone C, Ziv-Sokolovskaya N. Characterization and short-term outcome of potential coeliac disease in children. Journal of pediatric gastroenterology and nutrition. 2022;74(2 Supplement 2):165-6. |
| Kuja-Halkola 2017 | Kuja-Halkola R, Lebwohl B, Halfvarson J, Emilsson L, Magnusson PK, Ludvigsson JF. Birth weight, sex, and celiac disease: A nationwide twin study. Clinical epidemiology. 2017;9:567-77. |
| Lebwohl 2012 | Lebwohl B, Green PHR, Murray JA, Ludvigsson JF. Season of birth in a nationwide cohort of coeliac disease patients. Arch Dis Child. 2012;98(1):48-51. |
| Lebwohl 2013 | Lebwohl B, Blaser MJ, Ludvigsson JF, Green PHR, Rundle A, Sonnenberg A, et al. Decreased Risk of Celiac Disease in Patients With Helicobacter pylori Colonization. American journal of epidemiology. 2013;178(12):1721-30. |
| Lebwohl 2014 | Lebwohl B, Spechler SJ, Wang TC, Green PHR, Ludvigsson JF. Use of proton pump inhibitors and subsequent risk of celiac disease. Digestive and liver disease : official journal of the Italian Society of Gastroenterology and the Italian Association for the Study of the Liver. 2014;46(1):36-40. |
| Lehtinen 2016 | Lehtinen M, Eriksson T, Apter D, Hokkanen M, Natunen K, Paavonen J, et al. Safety of the human papillomavirus (HPV)-16/18 AS04-adjuvanted vaccine in adolescents aged 12-15 years: interim analysis of a large community-randomized controlled trial. Human vaccines and immunotherapeutics. 2016;12(12):3177‐85. |
| Leonard 2023 | Leonard MD, I.; Valitutti, F.; Burns, M.; Kenyon, V.; Zomorrodi, A.; Fasano, A. Uncovering The Role of Environmental Risk Factors in Celiac Disease Onset Journal of pediatric gastroenterology and nutrition. 2023;77:S476-S7. |
| Lewy 2009 | Lewy H, Meirson H, Laron Z. Seasonality of birth month of children with celiac disease differs from that in the general population and between sexes and is linked to family history and environmental factors. Journal of pediatric gastroenterology and nutrition. 2009;48(2):181-5. |
| Lionetti 2014 | Lionetti E, Castellaneta S, Francavilla R, Pulvirenti A, Tonutti E, Amarri S, et al. Introduction of Gluten, HLA Status, and the Risk of Celiac Disease in Children. The New England journal of medicine. 2014;371(14):1295-303. |
| Lionetti 2017 | Lionetti E, Castellaneta S, Francavilla R, Pulvirenti A, Catassi C, Sigenp Working Group of W, et al. Mode of Delivery and Risk of Celiac Disease: Risk of Celiac Disease and Age at Gluten Introduction Cohort Study. J Pediatr. 2017;184(NA):81-6 e2. |
| Lionetti 2020 | Lionetti E, Galeazzi T, Dominijanni V, Acquaviva I, Catassi GN, Iasevoli M, et al. Lower Level of Plasma 25-Hydroxyvitamin D in Children at Diagnosis of Celiac Disease Compared with Healthy Subjects: A Case-Control Study. The Journal of pediatrics. 2020;228(NA):132-7.e1. |
| Ludvigsson 2013 | Ludvigsson JF, Nordenskjold A, Murray JA, Olen O. A large nationwide population-based case-control study of the association between intussusception and later celiac disease. BMC Gastroenterol. 2013;13:89. |
| Ludvigsson 2013_1 | Ludvigsson JF, Reichenberg A, Hultman CM, Murray JA. A nationwide study of the association between celiac disease and the risk of autistic spectrum disorders. JAMA Psychiatry. 2013;70(11):1224-30. |
| Ludvigsson 2013_2 | Ludvigsson JF, Hadjivassiliou M. Can head trauma trigger celiac disease? Nation-wide case–control study. BMC neurology. 2013;13:1-6. |
| Lurz 2008 | Lurz E, Scheidegger UA, Spalinger J, Schöni MH, Schibli S. Clinical presentation of celiac disease and the diagnostic accuracy of serologic markers in children. Eur J Pediatr. 2008;168(7):839-45. |
| Maleki 2023 | Maleki M, MontazeriFar F, Payandeh A, Azadbakht Z. Prevalence of celiac disease and its related factors in children aged 2-6 years old: A case-control study. Nutrition and health. 2023;NA(NA):2601060231167456-026010602311674. |
| Mårild 2011 | Mårild K, Stephansson O, Montgomery S, Murray JA, Ludvigsson JF. Pregnancy Outcome and Risk of Celiac Disease in Offspring: A Nationwide Case-Control Study. Gastroenterology. 2011;142(1):39-45. |
| Marild 2013 | Marild K, Ye W, Lebwohl B, Green PHR, Blaser MJ, Card T, et al. Antibiotic exposure and the development of coeliac disease: a nationwide case-control study. BMC Gastroenterol. 2013;13:109. |
| Mårild 2013_1 | Mårild K, Stephansson O, Grahnquist L, Cnattingius S, Söderman G, Ludvigsson JF. Down Syndrome Is Associated with Elevated Risk of Celiac Disease: A Nationwide Case-Control Study. The Journal of pediatrics. 2013;163(1):237-42. |
| Mårild 2016 | Mårild K, Størdal K, Hagman A, Ludvigsson JF. Turner Syndrome and Celiac Disease: A Case-Control Study. Pediatrics. 2016;137(2):1-8. |
| Miranda 2017 | Miranda S, Chaignot C, Collin C, Dray-Spira R, Weill A, Zureik M. Human papillomavirus vaccination and risk of autoimmune diseases: a large cohort study of over 2 million young girls in France. Vaccine. 2017;35(36):4761-8. |
| Moos 2020 | Moos C, Duus KS, Frederiksen P, Heitmann BL, Andersen V. Exposure to the danish mandatory vitamin D fortification policy in prenatal life and the risk of developing coeliac disease—the importance of season: a semi ecological study. Nutrients. 2020;12(5):1243. |
| Moyer 2018 | Moyer K, Crespo M, Uli N, Sferra T. Celiac disease characteristics, prevalence, and incidence in children with type 1 diabetes mellitus. Journal of pediatric gastroenterology and nutrition. 2018;67(Supplement 1):S250. |
| Naddei 2022 | Naddei R, Di Gennaro S, Guarino A, Troncone R, Alessio M, Discepolo V. In a large Juvenile Idiopathic Arthritis (JIA) cohort, concomitant celiac disease is associated with family history of autoimmunity and a more severe JIA course: a retrospective study. Pediatr. 2022;20(1):31. |
| Namatovu 2014_2 | Namatovu F, Stromgren M, Ivarsson A, Lindgren U, Olsson C, Lindkvist M, et al. Neighborhood conditions and celiac disease risk among children in Sweden. Scandinavian journal of public health. 2014;42(7):572-80. |
| Narang 2017 | Narang M, Puri AS, Sachdeva S, Singh J, Kumar A, Saran RK. Celiac disease and Helicobacter pylori infection in children: Is there any Association? Journal of gastroenterology and hepatology. 2017;32(6):1178-82. |
| Norström 2020 | Norström F, Namatovu F, Carlsson A, Högberg L, Ivarsson A, Myléus A. Family socio‐economic status and childhood coeliac disease seem to be unrelated—A cross‐sectional screening study. Acta paediatrica (Oslo, Norway : 1992). 2020;110(4):1346-52. |
| Nusier 2010 | Nusier MK, Brodtkorb HK, Rein SE, Odeh A, Radaideh AM, Klungland H. Serological screening for celiac disease in schoolchildren in Jordan. Is height and weight affected when seropositive? Ital. 2010;36:1-6. |
| Odeh 2019 | Odeh R, Alassaf A, Gharaibeh L, Ibrahim S, Khdair Ahmad F, Ajlouni K. Prevalence of celiac disease and celiac-related antibody status in pediatric patients with type 1 diabetes in Jordan. Endocrine connections. 2019;8(6):780-7. |
| Oikarinen 2021 | Oikarinen M, Puustinen L, Lehtonen J, Hakola L, Simell S, Toppari J, et al. Enterovirus infections are associated with the development of celiac disease in a birth cohort study. Front. 2021;11:604529. |
| Patel 2018 | Patel B, Wi C-I, Hasassri ME, Divekar R, Absah I, Almallouhi E, et al. Heterogeneity of asthma and the risk of celiac disease in children. Allergy and asthma proceedings. 2018;39(1):51-8. |
| Pelayo 2020 | Pelayo SL, Sánchez MP, Pérez PD, Buendía DG, Sainz-Pardo EP, Santa-Cruz BV, et al. Infections in early life as risk factor for coeliac disease. An Pediatr (Engl Ed). 2020;94(5):293-300. |
| Peters 2001 | Peters U, Schneeweiss S, Trautwein EA, Erbersdobler HF. A case-control study of the effect of infant feeding on celiac disease. Annals of nutrition and metabolism. 2001;45(4):135-42. |
| Prosperi 2021 | Prosperi M, Santocchi E, Brunori E, Cosenza A, Tancredi R, Muratori F, et al. Prevalence and Clinical Features of Celiac Disease in a Cohort of Italian Children with Autism Spectrum Disorders. Nutrients. 2021;13(9). |
| Renata 2022 | Renata A, Ilaria C, Martina G, Donatella C, Fortunata C, Marianna M, et al. Gluten consumption and inflammation affect the development of celiac disease in at-risk children. Sci. 2022;12(1):5396. |
| Roberts 2009 | Roberts SE, Williams JG, Meddings D, Davidson R, Goldacre MJ. Perinatal risk factors and coeliac disease in children and young adults: a record linkage study. Aliment Pharmacol Ther. 2009;29(2):222-31. |
| Roman 2010 | Roman E. Environmental factors in coeliac disease: A nationwide case-control study (Repac Study). Journal of pediatric gastroenterology and nutrition. 2010;50(SUPPL. 2):E74-E5. |
| Rutz 2002 | Rutz R, Ritzler E, Fierz W, Herzog D. Prevalence of asymptomatic celiac disease in adolescents of eastern Switzerland. Swiss medical weekly. 2002;132(3-4):43-7. |
| Saadah 2012 | Saadah OI, Al-Agha AE, Al Nahdi HM, Bokhary RY, Bin Talib YY, Al-Mughales JA, et al. Prevalence of celiac disease in children with type 1 diabetes mellitus screened by anti-tissue transglutaminase antibody from Western Saudi Arabia. Saudi Med J. 2012;33(5):541-6 |
| Saari 2015 | Saari A, Harju S, Mäkitie O, Saha M-T, Dunkel L, Sankilampi U. Systematic Growth Monitoring for the Early Detection of Celiac Disease in Children. Jama, Pediatr. 2015;169(3):1525-NA. |
| Sadiq 2022 | Sadiq A, Khan J, Ullah I, Basharat N, Ali S, Din AU, et al. Seroprevalence of Anti-tTg-IgA among Symptomized Celiac Disease Patients and Their Correlation with Rotavirus Infection. Biomed Res Int. 2022;2022:6972624. |
| Savilahti 2018 | Savilahti EM, Ilonen J, Kukkonen AK, Savilahti E, Kuitunen M. Celiac Disease by the Age of 13 Years Is Not Associated With Probiotics Administration in Infancy. Journal of Pediatric Gastroenterology and Nutrition. 2018;66(6):937-40. |
| Schaub 2015 | Schaub RL, Escalante M, Haymond M, Redondo MJ. Celiac autoimmunity (CA) and celiac disease (CD) in children with type 1 diabetes (T1D). Diabetes. 2015;64(SUPPL. 1):A18. |
| Segerstad 2018 | Hård af Segerstad EM, Lee H-S, Andrén Aronsson C, Yang J, Uusitalo U, Sjöholm I, et al. Daily intake of milk powder and risk of celiac disease in early childhood: a nested case-control study. Nutrients. 2018;10(5):550. |
| Selimoğlu 2013 | Selimoglu MA, Kelles M, Erdem T, Ertekin V, Karabiber H, Selimoglu E. Craniofacial features of children with celiac disease. Eur J Gastroenterol Hepatol. 2013;25(10):1206-11. |
| Simre 2016 | Simre K, Uibo O, Peet A, Tillmann V, Kool P, Hamalainen A-M, et al. Exploring the risk factors for differences in the cumulative incidence of coeliac disease in two neighboring countries: the prospective DIABIMMUNE study. Digestive and liver disease : official journal of the Italian Society of Gastroenterology and the Italian Association for the Study of the Liver. 2016;48(11):1296-301. |
| Simre 2019 | Simre K, Uibo O, Peet A, Puustinen L, Oikarainen S, Tamminen K, et al. Early-life exposure to common virus infections did not differ between coeliac disease patients and controls. Acta paediatrica (Oslo, Norway : 1992). 2019;108(9):1709-16. |
| Stagi 2005 | Stagi S, Giani T, Simonini G, Falcini F. Thyroid function, autoimmune thyroiditis and coeliac disease in juvenile idiopathic arthritis. Rheumatology (Oxford, England). 2005;44(4):517-20. |
| Stahl 2020_1 | Stahl MG, Rasmussen CG, Dong F, Waugh K, Norris JM, Baxter J, et al. Mass Screening for Celiac Disease: The Autoimmunity Screening for Kids Study. The American journal of gastroenterology. 2020;116(1):180-7. |
| Stene 2006 | Stene LC, Honeyman MC, Hoffenberg EJ, Haas JE, Sokol RJ, Emery LM, et al. Rotavirus infection frequency and risk of celiac disease autoimmunity in early childhood: a longitudinal study. The American journal of gastroenterology. 2006;101(10):2333-40. |
| Tanpowpong 2013 | Tanpowpong P, Obuch JC, Jiang H, McCarty CE, Katz AJ, Leffler DA, et al. Multicenter study on season of birth and celiac disease: evidence for a new theoretical model of pathogenesis. The Journal of pediatrics. 2013;162(3):501-4. |
| Tanpowpong 2023 | Tanpowpong P, Li S, Espinola JA, Santos LC, James KE, Powe CE, et al. Pregnancy- and birth-related risk factors for the development of childhood celiac disease. Acta Paediatrica, International Journal of Paediatrics. 2023. |
| Tapia 2021 | Tapia G, Chuda K, Kahrs CR, Stene LC, Kramna L, Marild K, et al. Parechovirus Infection in Early Childhood and Association With Subsequent Celiac Disease. Am J Gastroenterol. 2021;116(4):788‐95. |
| Tjernberg 2014 | Tjernberg AR, Ludvigsson JF. Children with Celiac Disease Are More Likely to Have Attended Hospital for Prior Respiratory Syncytial Virus Infection. Digestive Diseases and Sciences. 2014;59(7):1502-8. |
| vanderPals 2014 | van der Pals M, Myléus A, Norström F, Hammarroth S, Högberg L, Rosén A, et al. Body mass index is not a reliable tool in predicting celiac disease in children. BMC Pediatr. 2014;14(1):1-6. |
| Walkowiak 2010 | Walkowiak J, Blask-Osipa A, Lisowska A, Oralewska B, Pogorzelski A, Cichy W, et al. Cystic fibrosis is a risk factor for celiac disease. Acta biochimica Polonica. 2010;57(1):115-8. |
| Whyte 2013 | Whyte LA, Kotecha S, Watkins WJ, Jenkins HR. Coeliac disease is more common in children with high socio-economic status. Acta paediatrica (Oslo, Norway : 1992). 2013;103(3):289-94. |
| Zacay 2024 | Zacay G, Weintraub I, Regev R, Modan-Moses D, Levy-Shraga Y. Fracture risk among children and adolescents with celiac disease: a nationwide cohort study. Pediatr Res. 2024;95(1):386-92. |
| Study Group: Soroka University Medical Center | |
| Bendersky 2020 | Bendersky AG, Sheiner E, Zamstein O, Landau D, Levy A. 1118: Cesarean delivery for breech presentation as a risk-factor for long-term gastrointestinal morbidity of the offspring. American journal of obstetrics and gynecology. 2020;222(1 Supplement):S688. |
| Daniel 2019 | Daniel S, Kalansky A, Tsur A, Pinsk V, Ling G, Rannan R, et al. Seasonality of birth affects paediatric coeliac disease. Acta paediatrica (Oslo, Norway : 1992). 2019;108(3):529-34. |
| Davidesko 2021 | Davidesko S, Alioshin A, Walfisch A, Wainstock T, Yerushalmi B, Sheiner E. Mode of delivery and long-term gastrointestinal-related hospitalization of the offspring. The journal of maternal-fetal & neonatal medicine : the official journal of the European Association of Perinatal Medicine, the Federation of Asia and Oceania Perinatal Societies, the International Society of Perinatal Obstetricians. 2021:1-10. |
| Gutvirtz 2024 | Gutvirtz G, Pariente G, Wainstock T, Sheiner E. 1013 Preterm delivery and the risk for childhood celiac disease of the offspring. Am J Obstet Gynecol. 2024;230(1):S534.  37. |
| Karur 2021 | Karur O, Gutvirtz G, Wainstock T, Sheiner E. Maternal prenatal smoking and long-term gastrointestinal morbidity of the offspring: A population-based cohort analysis. Reproductive toxicology (Elmsford, NY). 2021;103:133-8. |
| Pariente 2019 | Pariente G, Wainstock T, Sheiner E. 165: Maternal celiac disease and long-term gastrointestinal morbidity of the offspring. American journal of obstetrics and gynecology. 2019;220(1 Supplement):S124-S5. |
| Steiner 2019 | Steiner N, Wainstock T, Sheiner E, Segal I, Landau D, Walfisch A. Small for gestational age as an independent risk factor for long-term pediatric gastrointestinal morbidity of the offspring. The journal of maternal-fetal & neonatal medicine : the official journal of the European Association of Perinatal Medicine, the Federation of Asia and Oceania Perinatal Societies, the International Society of Perinatal Obstetricians. 2019;32(9):1407-11. |
| Yoles 2018 | Yoles I, Wainstock T, Sheiner E, Landau D, McFarlane C, Walfisch A. The association between Maternal GroupB Streptococcus colonization and offspring Gastro-Intestinal morbidity. American journal of obstetrics and gynecology. 2018;218(1 Supplement 1):S506-S7. |
| Study Group: The Environmental Determinants of the Diabetes in the Young (TEDDY) | |
| Aronsson 2015 | Aronsson CA, Lee H-S, Liu E, Uusitalo U, Hummel S, Yang J, et al. Age at gluten introduction and risk of celiac disease. Pediatrics. 2015;135(2):239-45. |
| Aronsson 2015_1 | Aronsson CA, Lee H-S, Koletzko S, Uusitalo U, Yang J, Virtanen SM, et al. Effects of Gluten Intake on Risk of Celiac Disease: A Case-Control Study on a Swedish Birth Cohort. Clinical gastroenterology and hepatology : the official clinical practice journal of the American Gastroenterological Association. 2015;14(3):403-9. |
| Aronsson 2019 | Aronsson CA, Lee H-S, Segerstad EMHa, Uusitalo U, Yang J, Koletzko S, et al. Association of gluten intake during the first 5 years of life with incidence of celiac disease autoimmunity and celiac disease among children at increased risk. JAMA. 2019;322(6):514-23. |
| Aronsson 2021 | Andrén Aronsson C, Liu X, Norris JM, Uusitalo U, Butterworth MD, Koletzko S, et al. 25 (OH) D Levels in Infancy Is Associated With Celiac Disease Autoimmunity in At-Risk Children: A Case–Control Study. Frontiers in nutrition. 2021;8:720041. |
| Hagopian 2017 | Hagopian W, Lee H-S, Liu E, Rewers M, She J-X, Ziegler A-G, et al. Co-occurrence of Type 1 Diabetes and Celiac Disease Autoimmunity. Pediatrics. 2017;140(5). |
| HårdAfSegerstad 2022 | Hård Af Segerstad EM, Liu X, Uusitalo U, Agardh D, Andrén Aronsson C, Na NA. Sources of dietary gluten in the first 2 years of life and associations with celiac disease autoimmunity and celiac disease in Swedish genetically predisposed children: The Environmental Determinants of Diabetes in the Young (TEDDY) study. The American journal of clinical nutrition. 2022;116(2):394-403. |
| HårdAfSegerstad 2023 | Hård af Segerstad EM, Mramba LK, Liu X, Uusitalo U, Yang J, Norris J, et al. Associations of dietary patterns between age 9 and 24 months with risk of celiac disease autoimmunity and celiac disease among children at increased risk. The American Journal of Clinical Nutrition. 2023;118(6):1099-105. |
| Kemppainen 2017 | Kemppainen KM, Lynch KF, Liu E, Lonnrot M, Simell V, Briese T, et al. Factors That Increase Risk of Celiac Disease Autoimmunity After a Gastrointestinal Infection in Early Life. Clinical gastroenterology and hepatology : the official clinical practice journal of the American Gastroenterological Association. 2017;15(5):694-702.e5. |
| Kemppainen 2017_1 | Kemppainen KM, Vehik K, Lynch K, Larsson HE, Canepa R, Simell V, et al. Association Between Early-Life Antibiotic Use and the Risk of Islet or Celiac Disease Autoimmunity. Jama, Pediatr. 2017;171(12):1217-25. |
| Koletzko 2018 | Koletzko S, Lee H-S, Beyerlein A, Aronsson CA, Hummel M, Liu E, et al. Cesarean Section on the Risk of Celiac Disease in the Offspring: The Teddy Study. Journal of Pediatric Gastroenterology and Nutrition. 2018;66(3):417-24.  51. |
| Laitinen 2018 | Laitinen A, Lee HS, Kurppa K, Lernmark A, Rewers M, Hagopian W, et al. The impact of genetic factors to familial predisposition of coeliac disease in the TEDDY study. Journal of pediatric gastroenterology and nutrition. 2018;66(Supplement 2):51-2. |
| Lindfors 2019 | Lindfors K, Lin J, Lee H-S, Hyöty H, Nykter M, Kurppa K, et al. Metagenomics of the faecal virome indicate a cumulative effect of enterovirus and gluten amount on the risk of coeliac disease autoimmunity in genetically at risk children: the TEDDY study. Gut. 2019;69(8):1416-22. |
| Liu 2014 | Liu E, Lee H-S, Aronsson CA, Hagopian W, Koletzko S, Rewers M, et al. Risk of pediatric celiac disease according to HLA haplotype and country. The New England journal of medicine. 2014;371(1):42-9. |
| Uusitalo 2015 | Uusitalo U, Lee H-S, Aronsson CA, Yang J, Virtanen SM, Norris JM, et al. Gluten consumption during late pregnancy and risk of celiac disease in the offspring: the TEDDY birth cohort. The American journal of clinical nutrition. 2015;102(5):1216-21. |
| Uusitalo 2019 | Uusitalo U, Andren Aronsson C, Liu X, Kurppa K, Yang J, Liu E, et al. Early Probiotic Supplementation and the Risk of Celiac Disease in Children at Genetic Risk. Nutrients. 2019;11(8). |
| Yang 2017 | Yang J, Tamura RN, Aronsson CA, Uusitalo U, Lernmark Å, Rewers M, et al. Maternal use of dietary supplements during pregnancy is not associated with coeliac disease in the offspring: The Environmental Determinants of Diabetes in the Young (TEDDY) study. The British journal of nutrition. 2017;117(3):466-72. |
| Study Group: Swedish Medical Birth Registry | |
| Adlercreutz 2015 | Adlercreutz EH, Wingren CJ, Vincente RP, Merlo J, Agardh D. Perinatal risk factors increase the risk of being affected by both type 1 diabetes and coeliac disease. Acta paediatrica (Oslo, Norway : 1992). 2015;104(2):178-84. |
| Namatovu 2014 | Namatovu F, Ivarsson A, Lindkvist M, Olsson C, Myleus A, Hogberg U, et al. Elective caesarean delivery is associated with increased celiac disease risk in boys, but not in girls. Gastroenterology. 2014;146(5 SUPPL. 1):S-468. |
| Namatovu 2016 | Namatovu F, Lindkvist M, Olsson C, Ivarsson A, Sandstrom O. Season and region of birth as risk factors for coeliac disease a key to the aetiology? Arch Dis Child. 2016;101(12):1114-8. |
| Namatovu 2016_2 | Namatovu F, Olsson C, Lindkvist M, Myléus A, Högberg U, Ivarsson A, et al. Maternal and perinatal conditions and the risk of developing celiac disease during childhood. BMC Pediatr. 2016;16(1):1-10. |
| Sandberg-Bennich 2002 | Sandberg-Bennich S, Dahlquist G, Kallen B. Coeliac disease is associated with intrauterine growth and neonatal infections. Acta paediatrica (Oslo, Norway : 1992). 2002;91(1):30-3. |
| Wingren 2011 | Wingren CJ, Bjorck S, Lynch KF, Ohlsson H, Agardh D, Merlo J. Coeliac disease in children: A social epidemiological study in Sweden. Acta Paediatrica, International Journal of Paediatrics. |
| Wingren 2012 | Wingren CJ, Agardh D, Merlo J. Revisiting the risk of celiac disease in children born small for gestational age: A sibling design perspective. Scand J Gastroenterol. 2012;47(6):632-9. |
| Wingren 2012_1 | Wingren CJ, Agardh D, Merlo J. Congenital anomalies and childhood celiac disease in Sweden. Journal of pediatric gastroenterology and nutrition. 2012;55(6):736-9. |
| Wingren 2012_2 | Wingren CJ, Agardh D, Merlo J. Sex differences in coeliac disease risk: A Swedish sibling design study. Digestive and Liver Disease. 2012;44(11):909-13. |
| Wingren 2012_3 | Wingren CJ, Agardh D, Merlo J. Acculturation and celiac disease risk in second-generation immigrants: a nationwide cohort study in Sweden. Scand J Gastroenterol. 2012;47(10):1174-80. |
| Study Group: BabyDiab |  |
| Hummel 2007 | Hummel S, Hummel M, Banholzer J, Hanak D, Mollenhauer U, Bonifacio E, et al. Development of autoimmunity to transglutaminase C in children of patients with type 1 diabetes: relationship to islet autoantibodies and infant feeding. Diabetologia. 2007;50(2):390-4. |
| Winkler 2019 | Winkler C, Jolink M, Knopff A, Kwarteng NA, Achenbach P, Bonifacio E, et al. Age, HLA, and sex define a marked risk of organ-specific autoimmunity in first-degree relatives of patients with type 1 diabetes. Diabetes Care. 2019;42(9):1684-91. |
| Study Group: Norwegian Mother and Child Cohort Study (MoBa) | |
| Emilsson 2015_1 | Emilsson L, Magnus MC, Stordal K. Perinatal risk factors for development of celiac disease in children, based on the prospective Norwegian Mother and Child Cohort Study. Clinical gastroenterology and hepatology : the official clinical practice journal of the American Gastroenterological Association. 2015;13(5):921-7. |
| Kahrs 2017 | Kahrs CR, Magnus MC, Stigum H, Lundin KEA, Størdal K. Early growth in children with coeliac disease: a cohort study. Arch Dis Child. 2017;102(11):1037-43. |
| Kahrs 2018 | Kahrs CR, Stordal K, Lund-Blix N, Marild K, Tapia G, Norris J, et al. Gluten intake in early childhood and risk of coeliac disease: A nationwide cohort study. Journal of pediatric gastroenterology and nutrition. 2018;66(Supplement 2):155-6. |
| Lund-Blix 2019 | Lund-Blix NA, Marild K, Tapia G, Norris JM, Stene LC, Stordal K. Gluten Intake in Early Childhood and Risk of Celiac Disease in Childhood: A Nationwide Cohort Study. Am J Gastroenterol. 2019;114(8):1299-306. |
| Lund-Blix 2020 | Lund-Blix NA, Tapia G, Marild K, Brantsaeter AL, Eggesbo M, Mandal S, et al. Maternal fibre and gluten intake during pregnancy and risk of childhood celiac disease: the MoBa study. Sci. 2020;10(1):16439. |
| Mårild 2015 | Mårild K, Kahrs CR, Tapia G, Stene LC, Størdal K. Infections and risk of celiac disease in childhood: a prospective nationwide cohort study. The American journal of gastroenterology. 2015;110(10):1475-84. |
| Marild 2017_1 | Marild K, Kahrs CR, Tapia G, Stene LC, Stordal K. Maternal Infections, Antibiotics, and Paracetamol in Pregnancy and Offspring Celiac Disease: A Cohort Study. Journal of pediatric gastroenterology and nutrition. 2017;64(5):730-6. |
| Marild 2017_2 | Marild K, Tapia G, Haugen M, Dahl SR, Cohen AS, Lundqvist M, et al. Maternal and neonatal vitamin D status, genotype and childhood celiac disease. PloS one. 2017;12(7):e0179080. |
| Mårild 2019_2 | Mårild K, Tapia G, Midttun Ø, Ueland PM, Magnus MC, Rewers M, et al. Smoking in pregnancy, cord blood cotinine and risk of celiac disease diagnosis in offspring. Eur J Epidemiol. 2019;34(7):637-49. |
| Størdal 2013 | Størdal K, Haugen M, Brantsæter AL, Lundin KEA, Stene LC. Association Between Maternal Iron Supplementation During Pregnancy and Risk of Celiac Disease in Children. Clinical gastroenterology and hepatology : the official clinical practice journal of the American Gastroenterological Association. 2013;12(4):624-31. |
| Størdal 2013_2 | Størdal K, White RA, Eggesbø M. Early Feeding and Risk of Celiac Disease in a Prospective Birth Cohort. Pediatrics. 2013;132(5):e1202-9. |
| Study Group: The Health Improvement Network (THIN) | |
| Tata 2015 | Tata LJ, Zingone F, Fleming KM, Card TR, Crooks CJ, Ciacci C, et al. Diagnosis of coeliac disease among children whose mothers have coeliac disease: A united kingdom general population-based cohort. United European gastroenterology journal. 2015;3(5 SUPPL. 1):A322. |
| Vajravelu 2018 | Vajravelu ME, Keren R, Weber DR, Verma R, De Leon DD, Denburg MR. Incidence and risk of celiac disease after type 1 diabetes: A population-based cohort study using the health improvement network database. Pediatr Diabetes. 2018;19(8):1422-8. |
| Zingone 2015 | Zingone F, West J, Crooks CJ, Fleming KM, Card TR, Ciacci C, et al. Socioeconomic variation in the incidence of childhood coeliac disease in the UK. Arch Dis Child. 2015;100(5):466-73. |
| Study Group: PreventCD | |
| Auricchio 2017 | Auricchio R, Cielo D, de Falco R, Galatola M, Bruno V, Malamisura B, et al. Respiratory Infections and the Risk of Celiac Disease. Pediatrics. 2017;140(4):NA-NA. |
| Auricchio 2020 | Auricchio R, Stellato P, Bruzzese D, Cielo D, Chiurazzi A, Galatola M, et al. Growth rate of coeliac children is compromised before the onset of the disease. Arch Dis Child. 2020;105(10):964-8. |
| Crespo-Escobar 2017 | Crespo-Escobar P, Mearin ML, Hervás D, Auricchio R, Castillejo G, Gyimesi J, et al. The role of gluten consumption at an early age in celiac disease development: a further analysis of the prospective PreventCD cohort study. The American journal of clinical nutrition. 2017;105(4):890-6. |
| CrespoEscobar 2018 | Crespo Escobar P, Castillejo G, Martinez-Ojinaga E, Donat E, Polanco I, Mearin ML, et al. Ten years of follow-up of the Spanish cohort of the European PreventCD study: the lessons learned. Revista espanola de enfermedades digestivas : organo oficial de la Sociedad Espanola de Patologia Digestiva. 2018;110(8):493-9. |
| Meijer 2022 | Meijer CR, Auricchio R, Putter H, Castillejo G, Crespo P, Gyimesi J, et al. Prediction models for celiac disease development in children from high-risk families: Data from the PreventCD cohort. Gastroenterology. 2022. |
| Vriezinga 2014 | Vriezinga SL, Auricchio R, Bravi E, Castillejo G, Chmielewska A, Escobar PC, et al. Randomized Feeding Intervention in Infants at High Risk for Celiac Disease. The New England journal of medicine. 2014;371(14):1304-15. |
| Study Group: Swedish Celiac Register | |
| Ivarsson 2002 | Ivarsson A, Hernell O, Stenlund H, Persson LA. Breast-feeding protects against celiac disease. The American journal of clinical nutrition. 2002;75(5):914-21. |
| Ivarsson 2003 | Ivarsson A, Persson LA, Nystrom L, Hernell O. The Swedish coeliac disease epidemic with a prevailing twofold higher risk in girls compared to boys may reflect gender specific risk factors. Eur J Epidemiol. 2003;18(7):677-84. |
| Ivarsson 2003_2 | Ivarsson A, Hernell O, Nyström L, Persson LÅ. Children born in the summer have increased risk for coeliac disease. Journal of Epidemiology & Community Health. 2003;57(1):36-9. |
| Myleus 2012_1 | Myleus A, Hernell O, Gothefors L, Hammarstrom M-L, Persson L-A, Stenlund H, et al. Early infections are associated with increased risk for celiac disease: an incident case-referent study. BMC Pediatr. 2012;12:194. |
| Myleus 2012_2 | Myleus A, Stenlund H, Hernell O, Gothefors L, Hammarstrom M-L, Persson L-A, et al. Early vaccinations are not risk factors for celiac disease. Pediatrics. 2012;130(1):e63-70. |
| Study Group: Danish Medical Birth Registry | |
| Andersen 2020 | Andersen V, Möller S, Jensen PB, Møller FT, Green A. Caesarean delivery and risk of chronic inflammatory diseases (inflammatory bowel disease, rheumatoid arthritis, coeliac disease, and diabetes mellitus): a population based registry study of 2,699,479 births in Denmark during 1973–2016. Clinical epidemiology. 2020:287-93. |
| Andersen 2021 | Andersen V, Pedersen AK, Möller S, Green A. Chronic inflammatory diseases–diabetes mellitus, rheumatoid arthritis, coeliac disease, crohn’s disease, and ulcerative colitis among the offspring of affected parents: A Danish population-based registry study. Clinical Epidemiology. 2021:13-20. |
| Crawley 2022 | Crawley C, Husby S. Early environmental risk factors for coeliac disease in adolescents, recruited from a population-based cohort. Journal of pediatric gastroenterology and nutrition. 2022;74(2 Supplement 2):142. |
| Jølving 2021 | Jølving LR, Anru PL, Nielsen J, Friedman S, Nørgård BM. The risk of chronic diseases and congenital malformations during childhood and adolescence after in utero exposure to thiopurines. Aliment Pharmacol Ther. 2021;54(8):1061-9. |
| Sevelsted 2015 | Sevelsted A, Stokholm J, Bonnelykke K, Bisgaard H. Cesarean section and chronic immune disorders. Pediatrics. 2015;135(1):e92-8. |
| Study Group: Friuli-Venezia Giulia Region | |
| Canova 2014 | Canova C, Zabeo V, Pitter G, Romor P, Baldovin T, Zanotti R, et al. Association of maternal education, early infections, and antibiotic use with celiac disease: a population-based birth cohort study in northeastern Italy. American journal of epidemiology. 2014;180(1):76-85. |
| Canova 2015 | Canova C, Pitter G, Ludvigsson JF, Romor P, Zanier L, Zanotti R, et al. Coeliac disease and asthma association in children: the role of antibiotic consumption. The European respiratory journal. 2015;46(1):115-22. |
| Study Group: Generation R | |
| Barroso 2018 | Barroso M, Beth SA, Voortman T, Jaddoe VWV, van Zelm MC, Moll HA, et al. Dietary Patterns After the Weaning and Lactation Period Are Associated With Celiac Disease Autoimmunity in Children. Gastroenterology. 2018;154(8):2087-96.e7. |
| Jansen 2014 | Jansen MAE, Tromp IIM, Jong JCK-d, Jaddoe VWV, Hofman A, Escher JC, et al. Infant feeding and anti-tissue transglutaminase antibody concentrations in the Generation R Study. The American journal of clinical nutrition. 2014;100(4):1095-101. |
| Jansen 2016 | Jansen MAE, van den Heuvel D, van der Zwet KVM, Jaddoe VWV, Hofman A, Escher JC, et al. Herpesvirus Infections and Transglutaminase Type 2 Antibody Positivity in Childhood: The Generation R Study. Journal of Pediatric Gastroenterology and Nutrition. 2016;63(4):423-30. |
| vanderVelde 2023 | van der Velde LA, Beth SA, Voortman T, van Zelm MC, Moll HA, Kiefte-de Jong JC. Anti-tissue transglutaminase antibodies (TG2A) positivity and the risk of vitamin D deficiency among children-a cross-sectional study in the generation R cohort. BMC Pediatr. 2023;23(1):286. |
| Study Group: All Babies in Southeast Sweden (ABIS) | |
| Ludvigsson 2005 | Ludvigsson JF, Ludvigsson J. Parental smoking and risk of coeliac disease in offspring. Scand J Gastroenterol. 2005;40(3):336-42. |
| Ludvigsson 2007 | Ludvigsson J. Socio-economic characteristics in children with coeliac disease. Acta paediatrica (Oslo, Norway : 1992). 2007;94(1):107-13. |
| Mårild 2010 | Mårild K, Frostell AS, Ludvigsson JF. Psychological stress and coeliac disease in childhood: a cohort study. BMC Gastroenterol. 2010;10(1):1-6. |
| Mårild 2014 | Mårild K, Ludvigsson J, Sanz Y, Ludvigsson JF. Antibiotic exposure in pregnancy and risk of coeliac disease in offspring: a cohort study. BMC Gastroenterol. 2014;14(1):1-7. |
| Welander 2010 | Welander A, Tjernberg AR, Montgomery SM, Ludvigsson J, Ludvigsson JF. Infectious disease and risk of later celiac disease in childhood. Pediatrics. 2010;125(3):e530-6. |
| White 2023 | White PA, Faresjö T, Jones MP, Ludvigsson J. Low maternal education increases the risk of Type 1 Diabetes, but not other autoimmune diseases: a mediating role of childhood BMI and exposure to serious life events. Sci. 2023;13(1):6166. |
| Study Group: Diabetes Autoimmunity Study in the Young (DAISY) | |
| Liu 2017 | Liu E, Dong F, Baron AE, Taki I, Norris JM, Frohnert BI, et al. High Incidence of Celiac Disease in a Long-term Study of Adolescents With Susceptibility Genotypes. Gastroenterology. 2017;152(6):1329-36.e1. |
| Marild 2018 | Marild K, Dong F, Lund-Blix N, Seifert J, Baron A, Waugh K, et al. Gluten intake and risk of celiac disease: Preliminary data from an at-risk birth cohort. Journal of pediatric gastroenterology and nutrition. 2018;66(Supplement 2):6-7. |
| Norris 2005 | Norris JM, Barriga KJ, Hoffenberg EJ, Taki I, Miao D, Haas JE, et al. Risk of Celiac Disease Autoimmunity and Timing of Gluten Introduction in the Diet of Infants at Increased Risk of Disease. JAMA. 2005;293(19):2343-51. |
| Stahl 2020_2 | Stahl MG, Dong F, Lamb MM, Waugh K, Taki I, Størdal K, et al. Childhood growth prior to screen-detected celiac disease: prospective follow-up of an at-risk birth cohort. Scand J Gastroenterol. 2020;55(11):1284-90. |

Table A3a

Predictive Factors for a diagnosis of Coeliac Disease, and the frequency in which they were reported in the included studies. This table presents all the remaining factors reported in <5 studies and therefore ineligible for inclusion in the meta-analysis.

| Rank | Factor | Total | Sig. | Rank | Factor | Total | Sig. |
| --- | --- | --- | --- | --- | --- | --- | --- |
| 32 | Deprivation | 4 | 3 | 60 | Hospitalized, any neonatal infection | 2 | 0 |
| 33 | Small for Gestational Age | 4 | 3 | 61 | HPV Vaccine | 2 | 0 |
| 34 | APGAR Score | 4 | 1 | 62 | Maternal Antibiotics | 2 | 0 |
| 35 | Autism Spectrum Disorder | 4 | 1 | 63 | Maternal Marital Status | 2 | 0 |
| 36 | Country | 4 | 1 | 64 | Milk Introduction | 2 | 0 |
| 37 | Atopy | 4 | 0 | 65 | Multiples | 2 | 0 |
| 38 | PPI Use | 3 | 3 | 66 | Adrenal Insufficiency | 1 | 1 |
| 39 | City | 3 | 2 | 67 | BCG Vaccination | 1 | 1 |
| 40 | Ear Infections | 3 | 2 | 68 | Craniofacial Features | 1 | 1 |
| 41 | Maternal Gluten Intake | 3 | 2 | 69 | Cystic Fibrosis | 1 | 1 |
| 42 | Age Starting Daycare | 3 | 1 | 70 | Family History of Atopy | 1 | 1 |
| 43 | Asthma | 3 | 1 | 71 | First Degree Relative with RA | 1 | 1 |
| 44 | First Degree Relative with T1DM | 3 | 1 | 72 | Food Given at Gluten Introduction | 1 | 1 |
| 45 | H. Pylori Infection | 3 | 1 | 73 | Fractures | 1 | 1 |
| 46 | Maternal BMI | 3 | 1 | 74 | GP Consultations | 1 | 1 |
| 47 | Probiotics | 3 | 1 | 75 | H2RA Use | 1 | 1 |
| 48 | Breech Presentation | 3 | 0 | 76 | Head Trauma | 1 | 1 |
| 49 | Maternal Diabetes | 3 | 0 | 77 | Herpesvirus Infection | 1 | 1 |
| 50 | Paternal Education | 3 | 0 | 78 | Hospitalized, neonatal GI Infection | 1 | 1 |
| 51 | Preeclampsia | 3 | 0 | 79 | Juvenile Idiopathic Arthritis | 1 | 1 |
| 52 | Family History Autoimmune Disease | 2 | 1 | 80 | Malformations | 1 | 1 |
| 53 | Maternal Country of Birth | 2 | 1 | 81 | Maternal Employment | 1 | 1 |
| 54 | Maternal Infections | 2 | 1 | 82 | Maternal Fibre Intake | 1 | 1 |
| 55 | Parental Country of Birth | 2 | 1 | 83 | Maternal Iron Supplementation | 1 | 1 |
| 56 | Rotavirus Vaccination | 2 | 1 | 84 | Mode of Gluten | 1 | 1 |
| 57 | Birth Height | 2 | 0 | 85 | Oats Introduction | 1 | 1 |
| 58 | Gestational Hypertension | 2 | 0 | 86 | Paternal Smoking | 1 | 1 |
| 59 | Gestational Weight Gain | 2 | 0 | 87 | Premature Rupture of Membranes | 1 | 1 |

Table A3b

(Continuation of Table A2a) Predictive Factors for a diagnosis of Coeliac Disease, and the frequency in which they were reported in the included studies. This table presents all the remaining factors reported in <5 studies and therefore ineligible for inclusion in the meta-analysis.

| Rank | Factor | Total | Sig. | Rank | Factor | Total | Sig. |
| --- | --- | --- | --- | --- | --- | --- | --- |
| 88 | Social Allowance | 1 | 1 | 118 | Maternal Latent TB | 1 | 0 |
| 89 | Sweden Region | 1 | 1 | 119 | Maternal Living Condition | 1 | 0 |
| 90 | Trisomy 21/Down Syndrome | 1 | 1 | 120 | Maternal Occupation | 1 | 0 |
| 91 | Turner Syndrome | 1 | 1 | 121 | Maternal Paracetamol | 1 | 0 |
| 92 | Williams Syndrome | 1 | 1 | 122 | Maternal Rh Status | 1 | 0 |
| 93 | Year of T1DM Diagnosis | 1 | 1 | 123 | Maternal Thiopurines | 1 | 0 |
| 94 | ADHD | 1 | 0 | 124 | Maternal UTI | 1 | 0 |
| 95 | Age at JIA Diagnosis | 1 | 0 | 125 | Maternal Vitamin D | 1 | 0 |
| 96 | Breath Holding Spells | 1 | 0 | 126 | Milk Powder Intake | 1 | 0 |
| 97 | Child Growth Indication C-Section | 1 | 0 | 127 | Milk Quantity | 1 | 0 |
| 98 | Child Iron Supplementation | 1 | 0 | 128 | Milk Type | 1 | 0 |
| 99 | Crowded Living | 1 | 0 | 129 | MMR Vaccine | 1 | 0 |
| 100 | Energy Intake | 1 | 0 | 130 | Neighbourhood | 1 | 0 |
| 101 | Enterovirus During Pregnancy | 1 | 0 | 131 | Neonatal Jaundice | 1 | 0 |
| 102 | Family History of Asthma | 1 | 0 | 132 | Outdoor Play | 1 | 0 |
| 103 | Family History of Thalassemia | 1 | 0 | 133 | Parental Stress | 1 | 0 |
| 104 | Flu Vaccination | 1 | 0 | 134 | Parental Worries | 1 | 0 |
| 106 | Forceps | 1 | 0 | 135 | Paternal Age | 1 | 0 |
| 107 | HiB Vaccination | 1 | 0 | 136 | Paternal Employment | 1 | 0 |
| 108 | Hydrolysed Formula | 1 | 0 | 137 | Paternal Living Condition | 1 | 0 |
| 109 | Infant Food Supplementation | 1 | 0 | 138 | Organic Pollutant Exposure | 1 | 0 |
| 110 | Intussusception | 1 | 0 | 139 | Pertussis Vaccination | 1 | 0 |
| 111 | Maternal Alcohol | 1 | 0 | 140 | Psychological Stress | 1 | 0 |
| 112 | Maternal Blood Group | 1 | 0 | 141 | Rice Introduction | 1 | 0 |
| 113 | Maternal Child Blood Group Incom. | 1 | 0 | 142 | Serious Life Event | 1 | 0 |
| 114 | Maternal GBS | 1 | 0 | 143 | Type of Water | 1 | 0 |
| 115 | Maternal Hypertension | 1 | 0 | 144 | UK Region | 1 | 0 |
| 116 | Maternal Indication for C-Section | 1 | 0 | 145 | UTI | 1 | 0 |
| 117 | Maternal Kidney Disease | 1 | 0 |  |  |  |  |

Table A4 Summary of predictive factors for the diagnosis of Coeliac Disease (reported in ≥5 included studies) and which studies reported the factor as significant (green check), or indeterminate significance, such as only in a subgroup (yellow exclamation mark), or nonsignificant (red x).

Table A5 Extracted Data on Age from studies reporting on it as a predictive factor.


Figure A1

Forest Plots for predictive factors 1-6 (numbering based on frequency of included studies reporting on each predictive factor). Sex, Breast Feeding, C-Section and Gluten Introduction plots show pooled odds ratio estimates. Height and Weight plots show standardized mean differences. Odds ratios and SMD calculated using HKSJ random effects model. Overall pooled estimate included for both HKSJ and DL random effects models on each plot.

Figure A2

Forest Plots for predictive factors 7-12 (numbering based on frequency of included studies reporting on each predictive factor). Odds ratios calculated using HKSJ random effects model. Overall pooled odds ratio estimate included for both HKSJ and DL random effects models on each plot.

Figure A3

Forest Plots for predictive factors 13-18 (numbering based on frequency of included studies reporting on each predictive factor). Odds ratios calculated using HKSJ random effects model. Overall pooled odds ratio estimate included for both HKSJ and DL random effects models on each plot.

Figure A4

Forest Plots for predictive factors 19-24 (numbering based on frequency of included studies reporting on each predictive factor). Odds ratios calculated using HKSJ random effects model. Overall pooled odds ratio estimate included for both HKSJ and DL random effects models on each plot.

Figure A5

Forest Plots for predictive factors 25-28 (numbering based on frequency of included studies reporting on each predictive factor). Odds ratios calculated using HKSJ random effects model. Overall pooled odds ratio estimate included for both HKSJ and DL random effects models on each plot.

Figure A6

Funnel Plots for predictive factors 1-6 (numbering based on frequency of included studies reporting on each predictive factor).

Figure A7

Funnel Plots for predictive factors 7-12 (numbering based on frequency of included studies reporting on each predictive factor).

Figure A8

Funnel Plots for predictive factors 13-18 (numbering based on frequency of included studies reporting on each predictive factor).

Figure A9

Funnel Plots for predictive factors 19-24 (numbering based on frequency of included studies reporting on each predictive factor).

Figure A10

Funnel Plots for predictive factors 25-28 (numbering based on frequency of included studies reporting on each predictive factor).
